# Impaired neurogenesis and neural progenitor fate choice in a human stem cell model of SETBP1 disorder

**DOI:** 10.1186/s13229-023-00540-x

**Published:** 2023-02-20

**Authors:** Lucia F. Cardo, Daniel C. de la Fuente, Meng Li

**Affiliations:** Neuroscience and Mental Health Innovation Institute, School of Medicine and School of Bioscience, Cardiff University, Hadyn Ellis Building, Maindy Road, Cardiff, CF24 4HQ UK

**Keywords:** CRISPR, Cas9 genome editing, Cortical development, Human pluripotent stem cell, In vitro differentiation, Neurogenesis, SETBP1, SETBP1 disorder, Wnt signaling

## Abstract

**Background:**

Disruptions of *SETBP1* (SET binding protein 1) on 18q12.3 by heterozygous gene deletion or loss-of-function variants cause SETBP1 disorder. Clinical features are frequently associated with moderate to severe intellectual disability, autistic traits and speech and motor delays. Despite the association of SETBP1 with neurodevelopmental disorders, little is known about its role in brain development.

**Methods:**

Using CRISPR/Cas9 genome editing technology, we generated a SETBP1 deletion model in human embryonic stem cells (hESCs) and examined the effects of SETBP1-deficiency in neural progenitors (NPCs) and neurons derived from these stem cells using a battery of cellular assays, genome-wide transcriptomic profiling and drug-based phenotypic rescue.

**Results:**

Neural induction occurred efficiently in all SETBP1 deletion models as indicated by uniform transition into neural rosettes. However, SETBP1-deficient NPCs exhibited an extended proliferative window and a decrease in neurogenesis coupled with a deficiency in their ability to acquire ventral forebrain fate. Genome-wide transcriptome profiling and protein biochemical analysis revealed enhanced activation of Wnt/*β*-catenin signaling in SETBP1 deleted cells. Crucially, treatment of the SETBP1-deficient NPCs with a small molecule Wnt inhibitor XAV939 restored hyper canonical *β*-catenin activity and restored both cortical and MGE neuronal differentiation.

**Limitations:**

The current study is based on analysis of isogenic hESC lines with genome-edited SETBP1 deletion and further studies would benefit from the use of patient-derived iPSC lines that may harbor additional genetic risk that aggravate brain pathology of SETBP1 disorder.

**Conclusions:**

We identified an important role for SETBP1 in controlling forebrain progenitor expansion and neurogenic differentiation. Our study establishes a novel regulatory link between SETBP1 and Wnt/*β*-catenin signaling during human cortical neurogenesis and provides mechanistic insights into structural abnormalities and potential therapeutic avenues for SETBP1 disorder.

**Supplementary Information:**

The online version contains supplementary material available at 10.1186/s13229-023-00540-x.

## Background

The cerebral cortex is the center of higher mental functions for humans and contains around 100 billion cells that account for about 76% of the brain's volume. Normal cortical development involves a set of highly complex and organized events, including neural stem cell proliferation, neuronal differentiation and appropriate positioning and interconnection of both excitatory and inhibitory neurons [1–3]. Abnormalities of cell proliferation or neurogenesis may cause malformations of the brain such as microcephaly, macrocephaly, or cortical dysplasia. Cortical malformations and aberrant neural circuitry have been implicated as an important cause of neurological disorders such as intellectual disability, autism, and developmental delay [4–7].

*SETBP1* gene is located at 18q12.3 and is associated with several neurodevelopmental disorders. SETBP1 haploinsufficiency due to heterozygous gene deletion or loss-of-function mutation causes SETBP1 disorder, a rare disorder with clinical features including expressive language impairment, intellectual disability, autistic-like traits, autism spectrum disorder (ASD), attention deficit hyperactivity disorder (ADHD), seizures, delayed motor skills and minor dysmorphic features amongst others [8–14]. The disorder is also known as SETBP1 haploinsufficiency disorder (SETBP1-HD) or Mental Retardation Dominant 29 (MIM #616,078). Its strong association with a phenotype of developmental delay with language disorder makes SETBP1 a new candidate gene for speech disorders [8, 12, 15, 16]. In contrast, point mutations of *SETBP1* result in SETBP1 gain-of-function due to impairment in its degradation [17] and causes a different disorder called Schinzel–Giedion syndrome (SGS). SGS is a severe multi-organ disorder characterized by distinctive facial features, profound neurodevelopmental and structural anomalies, and increased cancer risk [18–20]. Despite its clear association with several neurodevelopmental disorders, the function of SETBP1 in the developing brain remains unknown.

Human embryonic stem cells (hESCs) offer an infinite cell source for the generation of neural progenitors (NPCs) and neurons, and have proved to be an invaluable in vitro model for studying human neurodevelopment and associated neurological disorders. HESCs provide an isogenic model with defined genetic background in which disease-associated mutations can be generated using genome editing tools, such as the state-of-the-art CRISPR/Cas9 genome editing technology [21–23]. SETBP1 is expressed in the ventricular zone of the developing mouse telencephalon (http://www.eurexpress.org) and highly expressed in human neocortex (http://hbatlas.org). In order to study the effect of *SETBP1*-haploinsufficency on neurodevelopment we generated a *SETBP1* loss-of-function hESC model and investigated its impact on cortical neuronal differentiation. We found that *SETBP1*-deficient NPCs exhibited an extended proliferative window and a decrease in neurogenesis coupled with a deficiency in their ability to acquire ventral forebrain fate. Genome-wide transcriptome profiling and protein biochemical analysis indicated a novel regulatory link between *SETBP1* and Wnt/B-catenin signaling during human cortical neurogenesis and provides mechanistic insights into the structural abnormalities and potential therapeutic avenues for *SETBP1* disorder. Our data thus raise interesting questions as to the impact of these early developmental deficits on cortical neuronal differentiation.

## Methods

### CRISPR/Cas9 genome editing

**Fig. 1:**
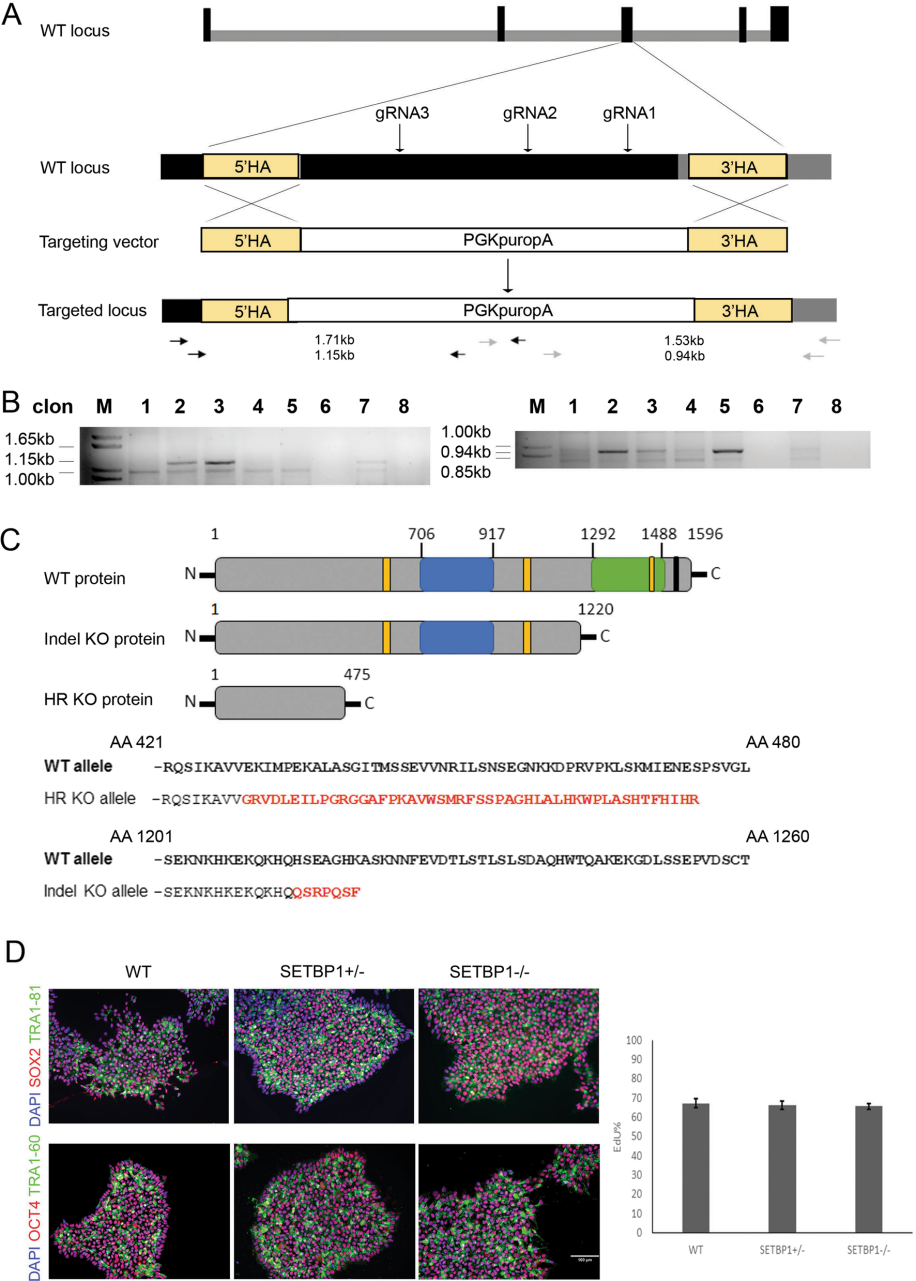
Generation of the SETBP1-deficient hESC lines.** A** Schematic illustration of the wild type (WT) SETBP1 locus and targeting strategy. Exons are shown in black and introns in grey. The three gRNAs targeting exon 4 are indicated in arrows. The homologous arms (HA) corresponding to exon 4 and part of intron 4/5 are indicated in yellow, which are flanked by a PGKpuropA selection cassette in the targeting vector. The positions of the two Nested PCR primer pairs for screening homologous recombination (HR) at the 5′ and 3′ are indicated in black and grey arrows, respectively, with the predicted PCR amplicon size indicated. **B** Agarose gels showing the final Nested PCR amplicon from the WT and targeted clones (lanes 2, 3, 7); left, 5′ primers; right 3′ primers. **C** Schematic illustration of the WT and predicted SETBP1 protein product from each of the two *SETBP1* mutant allele. Yellow = AT hook domains, blue = SKI homologous region, green = SET binding domain, black = repeat domain. Amino acid sequence alignment of WT SETBP1 protein versus HR and indel allele are shown. Amino acids in red indicate the sequence different from the WT prior to the stop codon. **D** Representative immunostaining of WT, SETBP1+/- and SETBP1-/- clones for pluripotency markers SOX2 (red), TRA1-81 (green), TRA1-60 (green) and OCT3/4 (red) with DAPI counterstain. Bar graph shows the proportion of EdU^+^ in WT, SETBP1+/- and SETBP1-/- hESCs (*P* > 0.05). Data presented as mean ± s.e.m of two independent experiments. AA: amino acid; N: NH2 terminal; C:COOH terminal. Scale bar: 50uM

Guide RNAs (gRNAs) were designed to target exon 4 of the human *SETBP1* gene using two independent CRISPR gRNA design tools: Atum CRISPR gRNA (former DNA2.0 https://www.atum.bio/eCommerce/cas9/input) and the CRISPR Design Tool (http://crispr.mit.edu) to minimize the risk of off-target effects of Cas9 nuclease. gRNA1 5′-TGTGGCCGGCTTCGCTGTGCTGG, gRNA2 5′-GGAGGTCATCGCGGTTTTGCAGG gRNA3 5′-TGAAATTTCATCTCGCTCATGGG. All gRNAs were synthesized as oligonucleotides and cloned into the pSpCas9(BB)-2A-GFP plasmid (px458, Addgene) following the protocol of Ran et al. [23]. A donor template (gene targeting) vector for homologous recombination was constructed that contains a PGK-puro-pA selection cassette flanked by a 502 bp 5′ homologous arm corresponding to part of exon 4 and 551 bp 3′ homologous arm in intron 4/5. All three gRNA target sites are located within the 2705 bp region between the two homologous arms (Fig. 1A). HESCs were transfected with a total of 4ug DNA in a ratio 2:3 (gRNAs:donor template) using the Amaxa P3 Primary Cell 4D-Nucleofector Kit (Lonza). Puromycin was added 3 days after electroporation at a concentration of 0.5ug/ml, drug resistant hESC colonies were picked one week later and expanded clonally. Genotyping was done by Nested PCR (Fig. 1A, primers used are provided in Additional file 1: Table S1) followed by Sanger sequencing of candidate mutant PCR product. Only SETBP1 heterozygous lines were generated in the first round of targeting (Fig. 1B lanes 2, 3 and 7 for mutant clones confirmed with 5′ and 3′ PCRs).

To generate SETBP1 homozygous clones, we subjected the heterozygous clone HET1 to a second round of targeting. The cells were nucleofected with the pSpCas9(BB)-2A-GFP plasmids in the absence of donor template plasmid. hESCs were subsequently passaged at clonal density and single clones manually picked and expanded for genetic screening. The targeting yield several clones carrying the original knockout allele plus a 5 bp deletion in the other allele (Additional file 2: Fig. S1B, C).

### Karyotyping

Sub-confluent hESCs cultures were treated with 0.1 μg/ml Demecolcine (Sigma D1925) for 1 h at 37 °C and then dissociated to a single cell suspension using Accutase (ThermoFisher) for 10 min at 37 oC. Cells were collected and washed twice with PBS by centrifugation for 4 min at 900 rpm. Cells were resuspended in 2 ml of PBS and 6 ml of 0.075 M KCl hypotonic solution was added to the tubes following incubation at 37 °C 15 min. Additional 4 ml of 0.075 KCl was added after incubation and cells were collected by centrifugation for 4 min at 900 rpm. The supernatant was removed leaving 300 μl to resuspend the cell pellet by flicking. 4 ml of pre-chilled (− 20 °C) methanol/acetic acid (3:1, VWR chemicals) was added dropwise and flicking to homogenize. Cell suspension was incubated for 30 min at room temperature. Cells were then centrifuged for 4 min at 900 rpm and resuspended with additional 4 ml of methanol/acetic acid. Cells were collected as previously and resuspended in 300 μl of methanol/acetic acid. Cell suspension was dropped onto a slide (pre-chilled and laid on angle) from a height of around 30 cm. Slides were air dried and chromosome spread was stained and mounted using a mix of mounting media with DAPI (1:3000). Images of chromosome spreads were obtained with an inverted microscope. Images were acquired at 100 × using the Leica Application Suite software and manually counted on ImageJ.

### HESC culture and neural differentiation

H7-WA07 (parental line) and genome-edited H7 derivatives (2 clones SETBP1 ± and 2 clones SETBP1-/-) were maintained on Matrigel-coated plates in Essential 8 media (TeSR-E8, Stemcell Technologies) [24]. All hESCs were passaged via manual dissociation using Gentle Cell Dissociation Reagent (Stemcell Technologies). For neural differentiation, hESCs were pre-plated on growth factor-reduced matrigel in TeSR-E8. When cells reached > 80% confluence, cortical differentiation was initiated by switching TeSR-E8 to DMEM-F12/Neurobasal (2:1) supplemented with N2 and B27 (referred to thereafter as N2B27). For the first 10 days, cultures were supplemented with SB431542 (10 µM, Tocris) and LDN-193189 (100 nM, StemGene). Cultures were passaged using EDTA firstly on day 10 at a ratio of 1:2 onto fibronectin-coated plates. The second and third split were performed at around day 20 and day 30, respectively, onto poly-D-lysine/laminin coated 24-well plates at a density of 125,000 cells/well. Retinol-free B27 was used for the first 25 days, followed by normal B27 from day 26 onwards. For Wnt/B-catenin inhibition, XAV939 (2 µM, SelleckChem) was added to the media for 10 days after the second split (day 10 to 20).

To differentiate the cells into a ventral MGE-like fate neural differentiation was initiated in the same way as described above with the addition of XAV939 (2 µM, SelleckChem) from day 0 to 10 and SHH-C24II (100 ng, 1845-SH-500 BioTechne) and Purmorphamine (0.5uM, 540,220–5 VWR) from day 10 to 20 [25, 26].

### Immunocytochemistry and EdU-labelling

Cultures were fixed with 4% (w/v) paraformaldehyde (PFA) and permeabilized with 0.1% (v/v) Triton X-100 in PBS. Following blocking with 1% (w/v) bovine serum albumin and 3% (v/v) donkey serum, cells were incubated with primary antibodies overnight at 4 °C. After three washes with PBS, cells were incubated with complementary Alexa Fluor-conjugated antibodies and counterstained with DAPI. All antibodies were diluted in PBS-T 1% donkey serum and incubated overnight at 4 degrees. Secondary antibodies were diluted (1:1000, Life Technologies) in PBS-T 1% donkey serum and incubated for 1 h at room temperature. The primary antibodies used are: Goat anti-OCT4, 1/500 (Santa Cruz), Goat anti-SOX2, 1/200 (Santa Cruz), Mouse anti-TRA1-60 and Mouse anti-TRA1-81 1/200 (both Millipore), Mouse anti-PAX6, 1/1000 (DHSB), Rabbit anti-OTX2, 1/300, Rabbit anti-NEUN, 1/500, Rabbit anti-GSH2 1/500 (both Millipore), Mouse anti-NESTIN, 1/300 (BD), Mouse anti-N-CAD, 1/100 (Life technologies), Mouse anti-ki67, 1/1000 (Leica biosystems), Rabbit anti-FAM107A, 1/100 (Atlas antibodies), Rabbit anti-PH3, 1/1000, Rabbit anti-TBR1, 1/500, Rabbit anti-TBR2 (EOMES), 1/500, Rat anti-CTIP2, 1/500, Mouse anti-SATB2, 1/50, Rabbit anti-NKX2.1, 1/500 (all from Abcam).

For EdU labelling, cultures were dissociated and plated at 150,000 cells per well to normalize the starting number of cells across the cultures 24 h before the EdU pulse. Cultures were then incubated with 10 µM EdU (5-ethynyl-2′-deoxyuridine) in N2B27 media for 30 min before PFA fixation in the case of hESC cultures and 2 h for NPC cultures (day 34). EdU detection was carried out using the Click-iT EdU Alexa Fluor 488/555 imaging kit as per manufacturer instructions (Life Technologies). This was followed by immunocytochemistry for Ki67, PAX6, TBR2 and FAM107.

Images were acquired using a DMI600b inverted microscope (Leica Microsystems). Cell counting was carried out using CellProfiler [27] or FIJI [28] software to analyse a minimum of 5–10 randomly placed fields of view per stain. Data were collected from two to five independent differentiation runs, sample size (*n*) per experiment and genotype indicated in the figure legends.

### Quantitative RT-PCR (qPCR)

Total RNA was extracted using TRIzol (Invitrogen) and treated with TURBO DNA-free (Ambion). cDNA was generated using qScript cDNA synthesis kit (Quanta Biosciences). qPCR was performed with Mesa Green qPCR master mix (Eurogentec) with specific primers listed in (primers Additional file 1: Table S1). When possible, primers were designed to encompass exon-exon junctions. Cq values were normalized to *GAPDH* housekeeping reference gene and changes in expression level were calculated using the 2-∆∆CT method [29]. All data were obtained from 3 independent differentiations with PCR carried out in 2 independent runs each with three technical replicates. All PCRs were run on a QuantStudio Real-time PCR machine (Applied Biosystems).

### Flow cytometry

Cultured CNPs at differentiation day 34 were dissociated in Accutase (ThermoFisher) for 10 min at 37 oC, then washed in PBS and counted. Samples containing 3*10^6 cells were fixed in 70% EtOH at − 20 °C overnight. The cells were washed three times in DPBS and blocked in solution 1%BSA-3% donkey serum for 45 min. Following Mouse anti-NESTIN (1/300, BD) antibody in 1%BSA-1% donkey serum or mouse anti IgG, 2 h at RT. Cells were washed in PBS three times and incubated in secondary antibody (alexa488 1:1000, Life technologies) in 1%BSA-1% donkey serum for 1 h at RT. Cells were washed twice in PBS and incubated with RNaseA (200 µg/ml, ThermoFisher) for 30 min. Then centrifuged and treated with DAPI (0.3 µg/ml, ThermoFisher) for 10 min. Cells were washed twice in DPBS and resuspended in 0.5 ml to be filtrated to remove clumps (Corning™ Falcon™ Test Tube with Cell Strainer Snap Cap). The samples were analysed on a BD LSRFortessa cell analyser (BD Biosciences). Data was analyzed in FlowJo (BD Biosciences) and Statistical analysis was performed in SPSS (IBM).

### Growth curve study

Cells were seeded in triplicate at 50,000 cells/well onto poly-D-lysine/laminin coated 48 well plates at day 19. Retinol-free B27 was used for the first 25 days, followed by normal B27 from day 26 onwards. Cells were dissociated into single cells every 4–5 days and counted manually in a Neubauer chamber.

### RNA sequencing (RNAseq)

RNA was extracted and purified using the PureLink RNA Mini Kit (Thermofisher Scientific). Libraries were prepared using the TruSeq Stranded mRNA kit (Illumina) from 1 µg RNA extracted from 3 biological replicate samples each collected at 3 time points of differentiation (days 15, 21, and 34, *n* = 9 each for SETBP1-/- and the isogenic control cells, respectively). 75 bp paired-end sequencing was performed on a HiSeq 4000 sequencer (Illumina, USA) yielding 30–45 million reads per sample. Reads were mapped to the human genome (GRCh38) using Burrows-Wheeler Aligner algorithms [30] and individual gene read counts calculated using featureCounts [31]. DESeq2 was used to calculate differential gene expression with a cut-off of adjusted *p* value < 0.1 and a fold change (FC) > 1.5 [32]. Gene Ontology functional enrichment for biological processes was performed using DAVID (v6.8) for the top 1000 more significant genes (ranked by adjusted *p* value), with all the protein coding genes in our dataset as background [33]. Calculated *p* values were adjusted for multiple testing using the Benjamin-Hochberg correction. Raw sequence data files are publicly available from the NCBI Gene Expression Omnibus (GSE180185). Bioinformatics code used for differentially expressed genes (DEG) and gene ontology (GO) is publicly available in https://github.com/DanCF93/Cardo-et-al-2021.

### Comparative analysis with the developmental human brain atlas

Transcriptomic data from the BrainSpan Atlas of the Developing Human Brain was downloaded from www.brainspan.org. Each sample column was matched to the metadata and those without metadata were filtered out from analysis. The analysis was limited to the embryonic stages and genes with less than 1 FPKM in at least one sample were omitted from the analysis. To make values comparable between the BrainSpan and PSC-derived datasets, gene expression values were transformed into Z-scores. Finally, Spearman’s correlation was calculated between both datasets.

### Correlation with GWAS common variants using MAGMA

Summary statistic files for ASD and intelligence GWAS were downloaded from the psychiatric genomics consortium website www.med.unc.edu/pgc/. SNPs with an imputation value > 0.8 and a minor allele frequency > 1% were used for analysis. SNPs were mapped to the human genome with an annotation window of 5 and 10 kb upstream and downstream, respectively. Linkage disequilibrium between SNPs was calculated using the European panel of the 1000 genomes (phase 3) as raw genotype data. Duplicated rs IDs for the same SNP were dropped from analysis and synonymous IDs were accounted for using the SNPdb file.

The final raw file was used to calculate the enrichment amongst GO terms identified from the transcriptomic analysis of the SETBP1 mutant cells in MAGMA [34].

### Western blotting

Protein extraction was performed with RIPA buffer (NEB) in the presence of protease and phosphatase inhibitors (Sigma) and quantified using the Bio-Rad DC protein assay (Bio-Rad). Total protein lysates (10–15 ug) were resolved in Bolt bistris plus 4–12% gels (Life technologies). PVDF membranes were blocked for 2 h in 5% (w/v) BSA TBST buffer and the following primary antibodies diluted in blocking buffer were used: Mouse anti-GAPDH (1/5000, Abcam), mouse anti-B-catenin (sc7963, 1/1000, Santa Cruz Biotechnology), rabbit anti-P-Ser552 B-catenin (1/500, Cell signaling), rabbit anti-P-Ser675 B-catenin (1/500, Cell signaling), rabbit anti-LRP6 (1/500, Cell signaling), rabbit anti-P-LRP6 (1/500, Cell signaling). Incubation was performed overnight at 4C and primary antibodies were detected with anti-rabbit and anti-mouse HRP antibodies (Abcam) using the Luminata Crescendo Western HRP substrate (Millipore). Protein samples from 3 independent differentiations were analysed except the XAV treatment analysis where 2 rounds of differentiation were performed.

### Statistical analysis

Statistical analyses were performed using IBM SPSS 23 software. Student’s T test or Mann-Withney U test were used for comparisons between two groups. One-way ANOVA and Kruskal–Wallis Test were used for comparisons between three groups. Statistically significant differences were considered when *p* value ≤ 0.05. Two-tailed test was used unless indicated otherwise. Statistical methods involving RNAseq analysis were described above in the RNAseq section.

## Results

### Generation of human stem cell model of SETBP1-deficiency

A hESC model of *SETBP1* deletion was generated by CRISPR/Cas9 assisted gene targeting in the H7 hESC line. Three gRNAs that recognize non-overlapping sequences in exon 4 were co-transfected with the targeting vector. Independent hESC clones were screened for homologous recombination firstly by PCR using primer pairs immediately outside the 5′ and 3′ homology arms, respectively (Fig. 1A). Correct homologous recombination was verified in one allele of three independent clones by PCR followed by Sanger sequencing (Fig. 1B and Additional file 2: Fig. S1A). This gene targeting introduced an early stop codon in the mutant allele and is predicted to produce a truncated protein of 475 amino acid (aa) of the full 1596aa protein sequence (www.expasy.org. Figure 1C). One of the heterozygous lines (HET1) was subjected to a second round of editing using the same gRNAs without the donor plasmid, yielding several independent clones containing a 5 bp deletion in the other allele (Additional file 2: Fig. S1B). This 5 bp deletion introduced an early stop codon in the second allele and is predicted to produce a truncated protein of 1220 aa (Additional file 2: Fig. S1C). qPCR analysis of SETBP1 mRNA levels with primers binding downstream of the region targeted by the gRNAs (Forward in exon 4–5 junction and reverse in exon 5–6 junction) shows a reduction of at least 50% in the homozygous SETBP1 mutant clones (Additional file 2: Fig. S1D).

The isogenic wild-type (WT) parental line, two homozygous SETBP1 mutant hESC lines (Homo 1 and Homo 2, referred to together as SETBP1-/-), along with two heterozygous SETBP1 mutant hESC lines (Het 1 and Het 2, referred to together as SETBP1+/-), were chosen for subsequent studies. The SETBP1 edited lines exhibited characteristic pluripotent stem cell (PSC) morphology, expressed pluripotency markers OCT4 and SOX2, and grew at a similar rate to that of H7 (Fig. 1D). Moreover, they have normal karyotype (46, XX) in 73–82% of total metaphases analysed.

### Cortical neuronal differentiation is altered in SETBP1-deficient NPCs

**Fig. 2:**
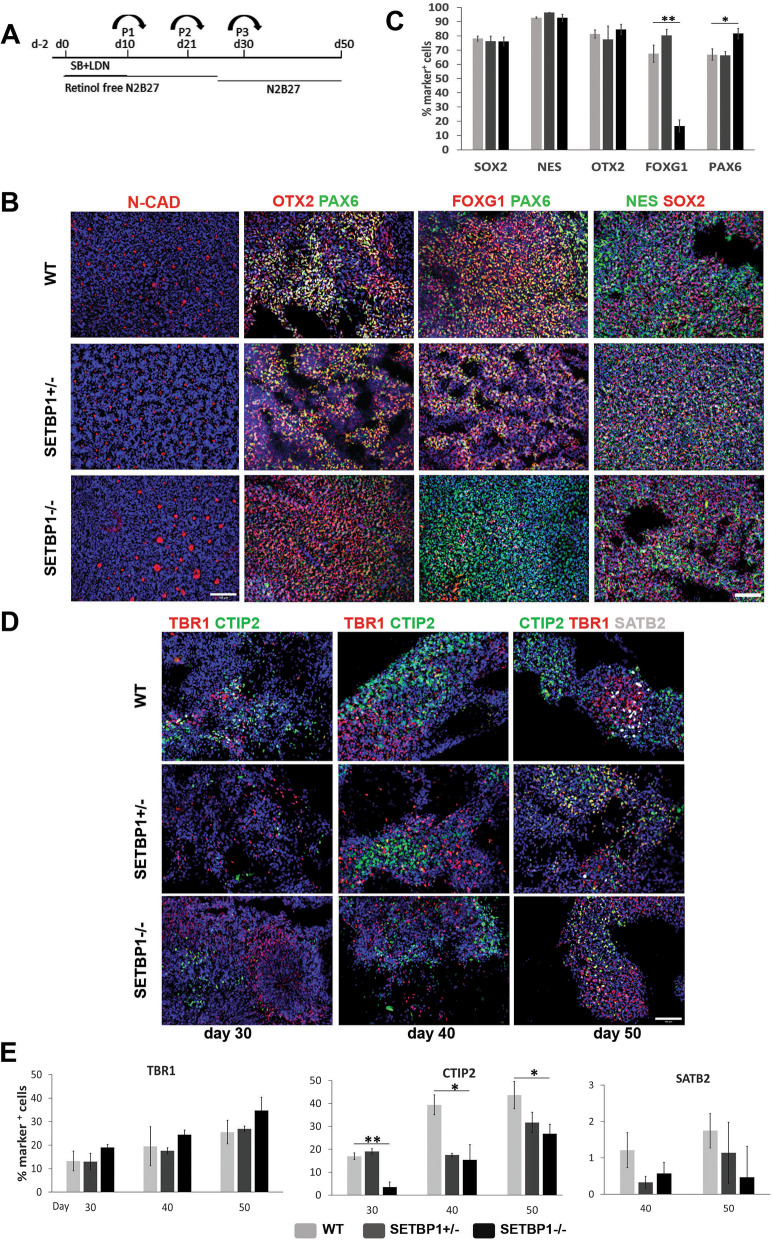
Neuronal differentiation is affected by loss of SETBP1 **A** Schematic representation of hESC cortical differentiation protocol. **B** Expression of NPC markers at day 18. Cultures were immunostained for N-cadherin (red) and DAPI (blue) showing the organization and size of the neural rosettes, PAX6 (green), OTX2 (red), NESTIN (NES, green), SOX2 (red) and FOXG1 (red). Dapi was used to label all nuclei. Scale bar: 100uM. **C** Quantitative data of marker expression presented as mean ± s.e.m for each genotype with a minimum of two independent experiments carried out per line and minimum of four per genotype (WT = 7, HET1 = 2, HET2 = 2, Homo1 = 6 and Homo 2 = 2). One-way ANOVA test with Bonferroni Post Hoc or Kruskal–Wallis non-parametric test, SOX2 *P* = 0.890, NES *P* = 0.499, OTX2 *P* = 0.606, FOXG1 *P* = 2.159E−06 (Post Hoc WT vs. SETBP1-/-, *p* = 0.000032; WT vs. SETBP1+/- , *p* = 0.331; SETBP1+/-  vs. SETBP1-/-, *p* = 3.77E−06), PAX6 *P* = 0.018 (Post Hoc WT vs. SETBP1-/-, *p* = 0.035; WT vs. SETBP1+/- , *p* = 1; SETBP1+/- vs. -/-, *p* = 0.052). (*p ≤ 0.05, **p ≤ 0.01, ***p ≤ 0.001). **D** Immunostaining of cortical layer markers TBR1 (layer VI) and CTIP2 (layers V–VI) at days 30, 40 and 50, respectively, and SATB2 (layers II–III) at day 50. Images representative of several independent experiments for each genotype **E** Quantitative analysis of the above. Data presented as mean ± s.e.m for each genotype with a minimum of two independent experiments carried out per line (WT = 5, Het1 = 2, 2 = 2, Homo1 = 3, and Homo2 = 2). One-way ANOVA test, Bonferroni Post Hoc; TBR1 *p* = 0.615 for day 30, 0.863 for day 40, and 0.585 for day 50; CTIP2 *P* = 0.004 for day 30 (Post Hoc WT vs. SETBP1-/-, *p* = 0.006; WT vs. SETBP1+/-, *p* = 1; SETBP1+/- vs. -/-, *p* = 0.007), 0.022 for day 40 (Post Hoc WT vs. SETBP1-/-, *p* = 0.05; WT vs. SETBP1+/-, *p* = 1; SETBP1+/- vs. -/-, *p* = 0.05), and 0.042 for day 50 (Post Hoc WT vs. SETBP1-/-, *p* = 0.0071; WT vs. SETBP1+/-, *p* = 1; SETBP1+/- vs. -/-, *p* = 0.182); SATB2 *P* = 0.491 for day 40, and 0.348 for day 50 (*p ≤ 0.05, **p ≤ 0.01, ***p ≤ 0.001). Scale bar: 100uM

The SETBP1 deficient and isogenic parental control hESCs were induced to differentiate toward cortical fate using a modified dual SMAD inhibition protocol as described previously (Fig. 2A) [24, 35]. Neural induction occurred efficiently in all three genotypes as indicated by the uniform transition into neural rosettes marked by N-cadherin (N-CAD) at their apical surface (Fig. 2B). To quantify the efficacy of neural induction and cortical fate commitment we performed antibody staining for markers of pan neural stem cell (SOX2, NESTIN), progenitors of telencephalon (FOXG1, OTX2) and dorsal telencephalon (PAX6) at day 18 (Fig. 2B–C). Indeed the vast majority of cells stained positive for SOX2, NESTIN, and OTX2 with comparable numbers of positive cells across the three genotypes (Fig. 2B–C), confirming normal neural induction of SETBP1-deficient hESCs. However, in the SETBP1-/- cultures we observed a ~ 70% reduction of FOXG1^+^ cells (*P* = 2.159E−06) and a moderate (~ 15%) increase in PAX6^+^ cells (*P* = 0.018) in comparison to the isogenic WT controls. No changes were found in the SETBP1+/- cultures. Consistent with immunostaining, RT-PCR analysis revealed a rapid induction of transcripts of a panel of pan-neural and forebrain-specific transcription factors and a decrease of *FOXG1* in the SETBP1-/- cultures compared to the WT (Additional file 3: Fig. S2A) although the level of *PAX6* appear similar until day 30 when an upregulation was observed in the SETBP1-/- cultures (Additional file 3: Fig. S2A, B).

We next examined the effect of SETBP1-deficiency on cortical neuronal production by immunostaining for a panel of commonly used cortical layer-specific markers (TBR1/layer VI, CTIP2/layers V–VI, SATB2/layers II–III) and a general neuronal marker (NeuN) at days 30, 40 and 50. We observed no significant change in TBR1^+^ neurons between the genotypes although the SETBP1-/- cultures exhibited a tendency of increase (Fig. 2D–E), which was observed at transcriptional level too (Additional file 3: Fig. S2B). The late born SATB2^+^ neurons were not detected at day 30 and were very low in number even at day 40 and 50 (~ 2%) in all cultures (Fig. 2D–E). However, evident decrease of CTIP2^+^ neurons was observed in SETBP1-/- cultures compared to the WT controls at all timepoints (ANOVA day 30 *P* = 0.004, day 40 *P* = 0.022, day 50 *P* = 0.042) (Fig. 2D–E). A decrease of CTIP2 transcripts was also observed (Additional file 3: Fig. S2B).

Reduced numbers of CTIP2^+^ cells were also observed in SETBP1+/- cultures from day 40, although statistical significance was only reached at day 40. Reduced numbers of NeuN^+^ cells were also detected in the SETBP1-/- at all three time points analysed, although statistical significance was only reached in the SETBP1+/- cultures for day 50 (ANOVA day 30 *P* = 0.012, day 40 *P* = 0.049, day 50 *P* = 0.009) (Additional file 4: Fig. S3A, B). Similarly, fewer MAP2^+^ cells can be observed in SETBP1-/- day 50 cultures while the proportion of NES^+^ cells was higher than the controls (Additional file 4: Fig. S3A). Together, these findings demonstrate a distorted neuronal production in SETBP1-/- cultures, while heterozygous deletion of SETBP1 had a milder effect.

### Loss of SETBP1 led to a bias toward neural progenitor proliferation

**Fig. 3:**
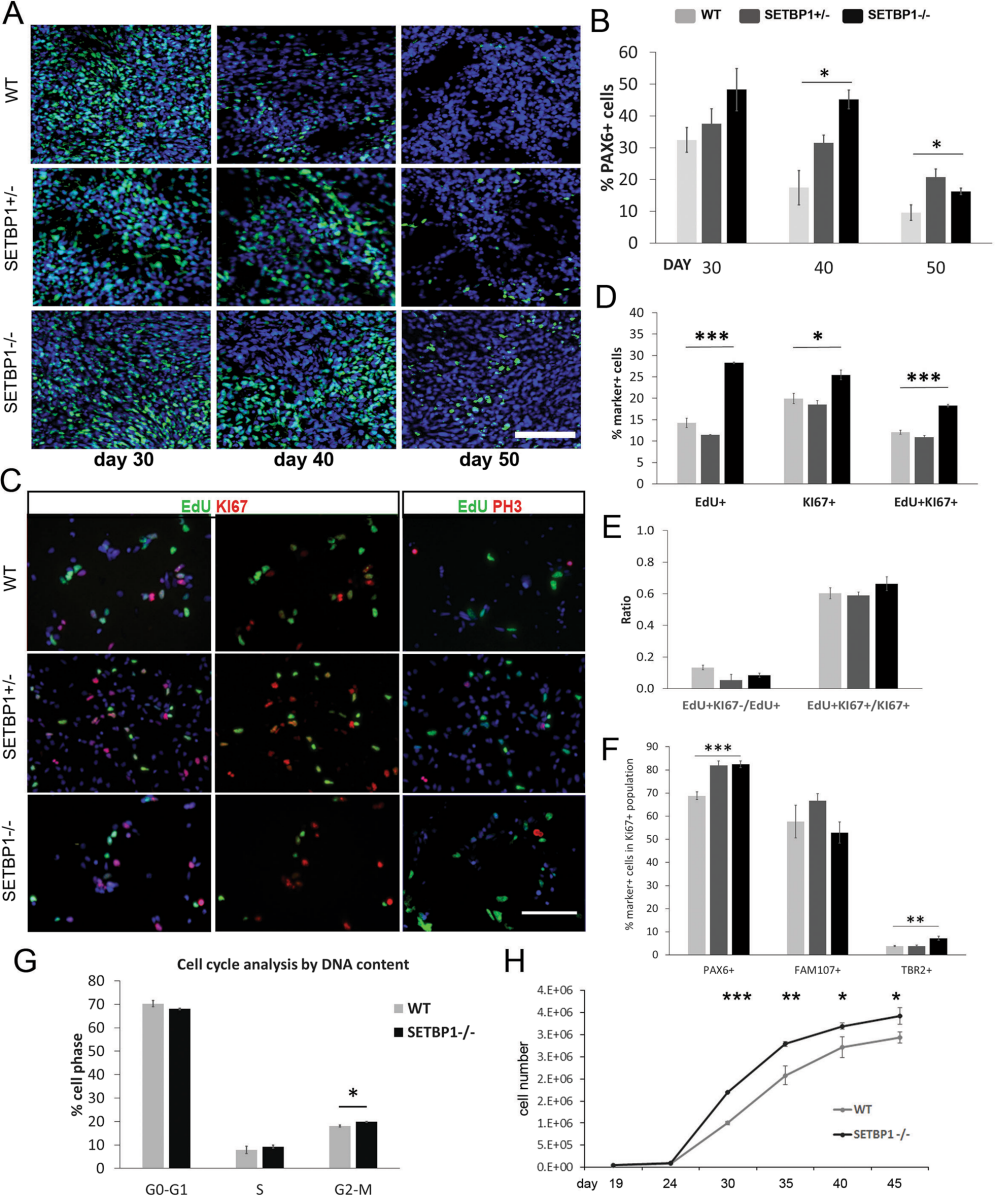
SETBP1 deficiency enhances cortical progenitor proliferation. **A** Cultures were immunostained for dorsal forebrain marker PAX6 (green) at days 30, 40 and 50. Dapi was used to label all nuclei. Scale bar: 100uM. **B** Quantitative data for PAX6 positive cells presented as mean ± s.e.m for each genotype with a minimum of two independent experiments carried out per line (WT = 5, HET2 = 2, KO1 = 3, and KO2 = 2). One-way ANOVA test with Bonferroni Post Hoc was used to compare the expression between the lines day 30 *P* = 0.181, day 40 *P* = 0.032 (Post Hoc WT vs. SETBP1-/-, *p* = 0.039; WT vs. SETBP1+/-, *p* = 0.289; SETBP1+/- vs. -/-, *p* = 0.385), day 50 *P* = 0.041 (Post Hoc WT vs. SETBP1-/-, *p* = 0.182; WT vs. SETBP1+/-, *p* = 0.050; SETBP1+/- vs. -/-, *p* = 0.473). **C** WT, SETBP1+/- (HET1) and SETBP1-/- (Homo1) day 35 cultures were immunostained for EdU (green), Ki67 (red), PH3 (red) and counterstained with DAPI (blue). Scale bar: 100uM. **D** Percentage of cells positive for EdU *P* = 4.87E−06 (Post Hoc WT vs. SETBP1-/-, *p* = 1.25E−05; WT vs. SETBP1+/- , *p* = 0.240; SETBP1+/- vs. -/-, *p* = 9.01E−06), Ki67 *P* = 0.016 (Post Hoc WT vs. SETBP1-/-, *p* = 0.038 WT vs. SETBP1+/- , *p* = 1; SETBP1+/- vs. -/-, *p* = 0.025), and EdU and Ki67 (cell cycle re-entry) *P* = 7.07E−06 (Post Hoc WT vs. SETBP1-/-, *p* = 1.68E−05 WT vs. SETBP1+/- , *p* = 0.354; SETBP1+/- vs. -/-, *p* = 1.38E−05). **E** Ratios of cell cycle exit and cell cycle length. Data presented as mean ± s.e.m from 3 independent wells with 6 random fields each. One-way ANOVA test (*P* = 0.055, *P* = 0.438). **F** Analysis of PAX6, TBR2 and FAM107A cycling progenitors in Ki67^+^ population. One-way ANOVA test with Bonferroni Post Hoc, PAX6^+^ *P* = 0.00097 (Post Hoc WT vs. SETBP1-/-, *p* = 0.001; WT vs. SETBP1+/- , *p* = 0.002; SETBP1 +/- vs. SETBP1-/-, *p* = 1), FAM107A^+^ *P* = 0.141, TBR2.^+^ *P* = 0.007 (Post Hoc WT vs. SETBP1-/-, *p* = 0.036; WT vs. SETBP1+/- , *p* = 0.996; SETBP1+/- vs. -/-, *p* = 0.01). **G** Cell cycle analysis by DNA content using Flow cytometry, % of cells in each of the cell cycle phases. Data presented as mean ± s.e.m of 2 independent experiments in triplicates. Student’s T test, one tail (G1-G0 *P* = 0.118, S *P* = 0.255, G2-M *P* = 0.029). **H** Growth curve analysis from day 19 to day 45 showing the increased population growth of the SETBP1-/- (Homo1) NPCs compared to the isogenic controls. Statistical significant differences were found from day 30 onwards (Student’s T test, *P* = 6.71E−05 for day 30, 0.015 for day35, 0.033 for day 40, and 0.050 for day 45). Data presented as mean ± s.e.m from 3 independent wells with two technical measurements. (*p ≤ 0.05, **p ≤ 0.01, ***p ≤ 0.001)

The reduced neuron production in the SETBP1-/- cultures could be due to an imbalance between progenitor proliferation versus terminal differentiation. We therefore determined PAX6^+^ cortical progenitor numbers during neurogenesis phase between days 30–50 by antibody staining. In the WT cultures, PAX6^+^ cell numbers drop from ~ 75% at day 18 to 30% at day 30 and 17% by day 40 (Fig. 2C, Fig. 3A, B). However, the rate of reduction was slower in the SETBP1-/- cultures where approximately 45% cells remained PAX6^+^ at day 40 (Fig. 3A, B), leading to a significant increase of PAX6^+^ NPCs in the SETBP1-deficient cultures. This finding is mirrored by sustained higher levels of *HES1* transcript in these cultures (Additional file 3: Fig. S2B).

The above observation suggests an increase in neural progenitor maintenance in the SETBP1-/- cultures. To investigate whether this is due to alterations in cell cycle profile upon loss of SETBP1, we performed EdU and Ki67 double labelling at day 34 (Fig. 3C–E). EdU is a thymidine analogue hence its incorporation marks cells in the S phase, while Ki67 is a protein present during all active phases of the cell cycle (G1, S, G2 and mitosis). The SETBP1+/- and WT cultures contained a similar number of EdU^+^ and Ki67^+^ cells (14.24 ± 1.10% vs. 11.42 ± 0.11% and 19.94 ± 1.21% vs. 18.52 ± 0.93%, respectively). However, significantly more EdU^+^ (28.27 ± 0.22%), Ki67^+^ (25.45 ± 1.14%) and EdU^+^Ki67^+^ cells (SETBP1-/-, 18.28 ± 0.56% vs. WT, 12.3 ± 1.7%) were detected in the SETBP1-/- cultures (ANOVA *P* = 4.88E−06, *P* = 0.0168, *P* = 7.08E−06) (Fig. 3C, D). The fraction of EdU^+^Ki67^−^ cells within the EdU^+^ population is often used as an index for cell cycle exit [36], we found that the ratio of EdU^+^Ki67^−^/EdU^+^ is lower in SETBP1-/- cultures than the SETBP1+/- and WT controls with a borderline *p* value (SETBP1-/-, 0.083 ± 0.013%, SETBP1+/- , 0.054 ± 0.036% WT, 0.133 ± 0.014%; ANOVA *P* = 0.055) (Fig. 3E), suggesting that SETBP1-/- NPCs were slow in exiting the cell cycle compared to their isogenic counterparts. The ratio of EdU^+^Ki67^+^/Ki67^+^ is inversely related to the length of cell cycle [37]. Consistent with an increase in proliferation, this ratio was slightly higher in SETBP1-/- cultures than the controls, indicating the former have shorter cell cycle (SETBP1-/-, 0.663 ± 0.043%, SETBP1+/- , 0.588 ± 0.021% WT, 0.603 ± 0.034%; ANOVA *P* = 0.438) (Fig. 3E). To further delineate the progenitor population targeted by SETBP1, we performed Ki67 co-staining with PAX6 (a marker for ventricular and outer radial glia), FAM107A (outer radial glia) and TBR2/EOMES (intermediate progenitor) (Fig. 3F). Within the cycling (Ki67^+^) progenitors, we found an enrichment of PAX6^+^ cells in the SETBP1-deficient lines. Moreover, there were more cycling TBR2^+^ intermediate progenitors in the SETBP1-/- cultures than the WT controls, providing another support on the compromised neurogenic state of the SETBP1-/- cultures.

To gain further insight into changes in cell cycle profile, we performed a flow cytometry-based cell cycle analysis (Fig. 3G). This assay identifies cells in three major phases of the cell cycle (G0/1, S and G2/M) based on their DNA content. Since the cellular defects observed were largely limited to the SETBP1-/- cultures, we focused on this genotype in the subsequent studies. Compared to the WT control, the SETBP1-/- cultures contained a higher percentage of NES + cells in S (9.24 ± 0.69% vs. 7.92 ± 1.52%) and G2/M phases (19.81 ± 0.18% vs. 18.07 ± 0.37%, *P* = 0.029) and fewer cells in G0/G1 (68.01 ± 0.31% vs. 70.24 ± 1.29%), although the number of cells in mitosis were similar as revealed by antibody staining for phosphorylated histone H3 (PH3) (Fig. 3C, Additional file 5: Fig. S4).

To investigate how altered cell cycle impact on the growth rate over time, we compared population growth of SETBP1-/- and WT cultures between day 19 and day 45. Consistent with EdU incorporation and cell cycle analysis, more cells were found in the SETBP1-/- cultures than the WT from day 30 onwards (*P* ≤ 0.05, Fig. 3H). Together, these findings demonstrate that SETBP1 deficiency leads to enhanced NPC division by regulating cell cycle.

### Loss of SETBP1 compromises ventral forebrain fate induction

**Fig. 4:**
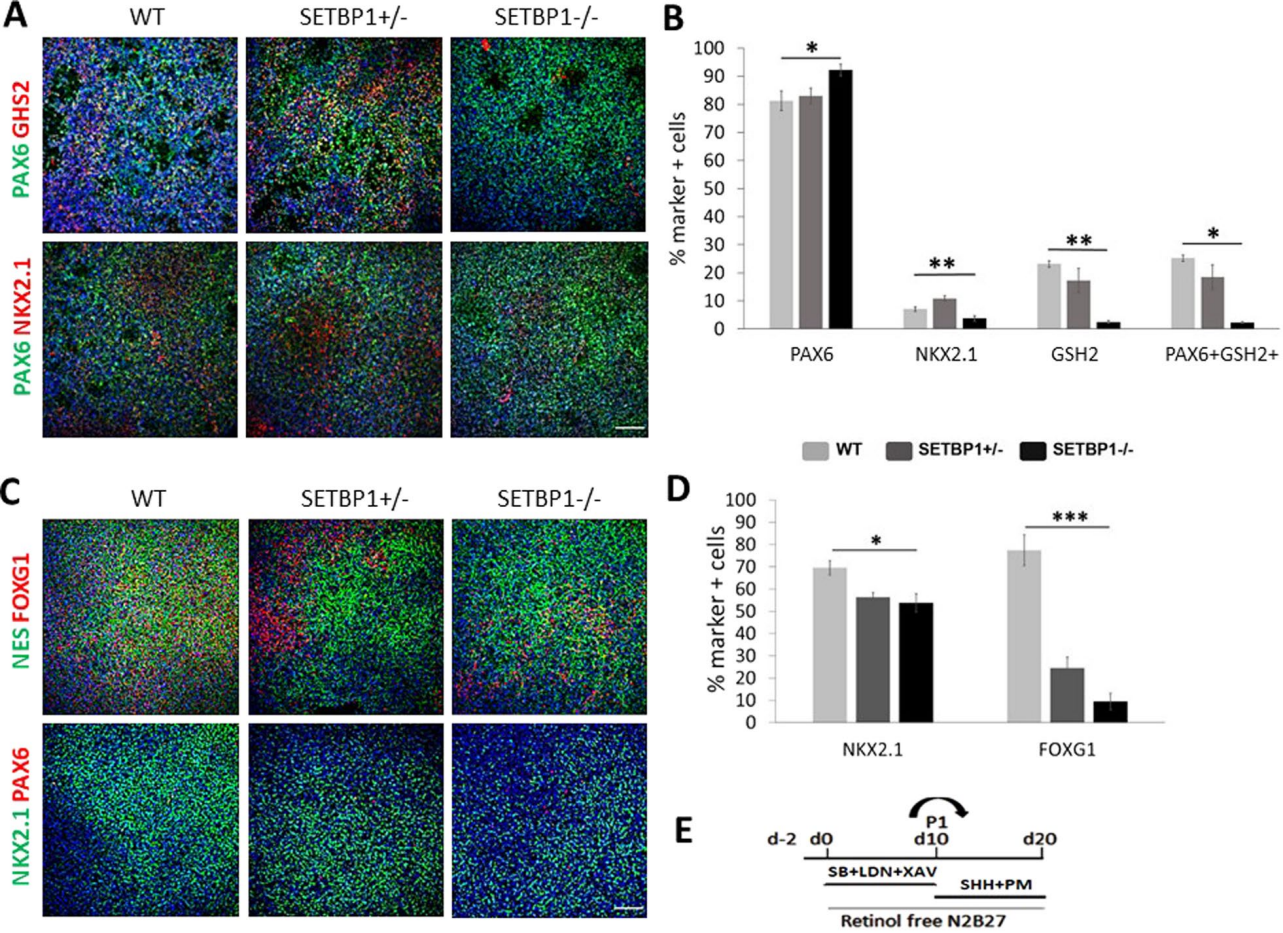
SETBP1 deficiency impairs acquisition of ventral forebrain identity. **A** Expression of dorsal forebrain marker PAX6 (green), dorso-ventral boundary forebrain marker GHS2 (red) and ventral marker NKX2.1 (red) in day 24 cortical NPCs. Dapi was used to label all nuclei. Scale bar: 100uM. **B** Quantitative data presented as mean ± s.e.m for each genotype with two biological replicas per line and genotype. One-way ANOVA or Kruskal–Wallis test: PAX6 *P* = 0.021 (Post Hoc WT vs. SETBP1-/-, *p* = 0.047 WT vs. SETBP1+/- , *p* = 0.912; SETBP1+/- vs. SETBP1-/-, *p* = 0.040), NKX2.1 *P* = 0.003 (Post Hoc WT vs. SETBP1-/-, *p* = 0.221 WT vs. SETBP1+/- , *p* = 0.138; SETBP1+/- vs. SETBP1-/-, *p* = 0.003), GSH2 *P* = 0.006 (Post Hoc WT vs. SETBP1-/-, *p* = 0.011 WT vs. SETBP1+/- , *p* = 0.803; SETBP1+/- vs. SETBP1-/-, *p* = 0.022), PAX6^+^GSH2^+^/PAX6^+^ *P* = 0.032 (Post Hoc WT vs. SETBP1-/-, *p* = 0.022 WT vs. SETBP1 +/- , *p* = 0.567; SETBP1+/- vs. SETBP1-/-, *p* = 0.036) (*p ≤ 0.05, **p ≤ 0.01, ***p ≤ 0.001). **C** Expression of pan-neural forebrain marker FOXG1 (red), neural radial glia marker NES (green), dorsal forebrain marker PAX6 (red), and ventral markers NKX2.1 (green) in ventrally derived (MGE) NPCs at day 20. Dapi was used to label all nuclei. Scale bar: 100uM. **D** Quantitative data presented as mean ± s.e.m for each genotype with three biological replicas per line and genotype. One-way ANOVA test: NKX2.1 *P* = 0.038 (Post Hoc WT vs. SETBP1-/-, *p* = 0.042 WT vs. SETBP1+/- , *p* = 0.098; SETBP1+/- vs. SETBP1, *p* = 1), FOXG1 *P* = 4.77E−06 (Post Hoc WT vs. SETBP1-/-, *p* = 3.86E−06 WT vs. SETBP1+/- , *p* = 4.8E−05; SETBP1+/- vs. SETBP1-/-, *p* = 0.103) (*p ≤ 0.05, **p ≤ 0.01, ***p ≤ 0.001). **E** Schematic representation of hESC ventral forebrain (MGE) differentiation protocol to generate ventral NPC population

Despite its pan-forebrain expression, loss of Foxg1 in mice preferentially impairs lateral and medial ganglionic eminence (LGE and MGE) formation [38] while overexpression of FOXG1 in human iPSCs leads to overproduction of MGE-derived neurons [39]. The dramatic reduction of FOXG1 expression in SETBP1 mutant cells prompted the examination of LGE and MGE progenitor content in the cortical cultures by antibody staining for GSH2 and NKX2.1 at day 24. In cortically differentiated WT control cultures ~ 5% cells stained positive to NKX2.1, a transcription factor with restricted expression in the MGE (Fig. 4A, B). The SETBP1-/- cultures contained even fewer NKX2.1^+^ cells (*P* = 0.003), implicating a decrease in MGE like progenitors. In the developing human brain, PAX6 expression extends beyond the cortex into the LGE [40]. To provide further insight into the increase of PAX6^+^ cells in the SETBP1-/- cultures, we performed double staining of PAX6 with GSH2, a transcription factor with restricted expression in the LGE and MGE (Fig. 4A, B). We detected around 20% of GSH2^+^ cells in the WT control cultures, nearly all of which co-expressed PAX6. However, only 2.5% GSH2^+^ and 2.3% GSH2^+^PAX6^+^ cells were found in the SETBP1-/- cultures (ANOVA *P* = 0.006, Kruskal–Wallis Test *P* = 0.032) (Fig. 4A, B), demonstrating a reduced capacity of the SETBP1-/- progenitors to adopt LGE fate. This finding also suggests that the observed increase of PAX6 + cells in the SETBP1-/- cultures are most likely of cortical identity.

Since the cortical protocol yielded limited number of ventral cell types, we also differentiated the WT and SETBP1-deficient hESCs using a MGE induction protocol (Fig. 4E). A reduction of NKX2.1^+^ cells was detected in both the SETBP1-/- and SETBP1+/- cultures compared to WT controls (ANOVA *P* = 0.038, Fig. 4C, D). We didn’t detect a change in the number of EdU^+^ and Ki67^+^ cells in the SETBP1 mutant cultures (Additional file 6: Fig. S5). However, a marked reduction of FOXG1^+^ cells was also observed in MGE differentiated SETBP1-/- cultures (*P* = 4.77E−06, Fig. 4C, D). Interestingly, unlike in the cortical cultures where no significant reduction of FOXG1^+^ were observed, a reduction was also detected in the SETBP1+/- cultures. These observations provide further support that SETBP1-deficiency compromise ventral forebrain fate induction.

### *Genome-wide transcriptome analysis identified Wnt/*β*-catenin signaling as a target of SETBP1 function*

**Fig. 5:**
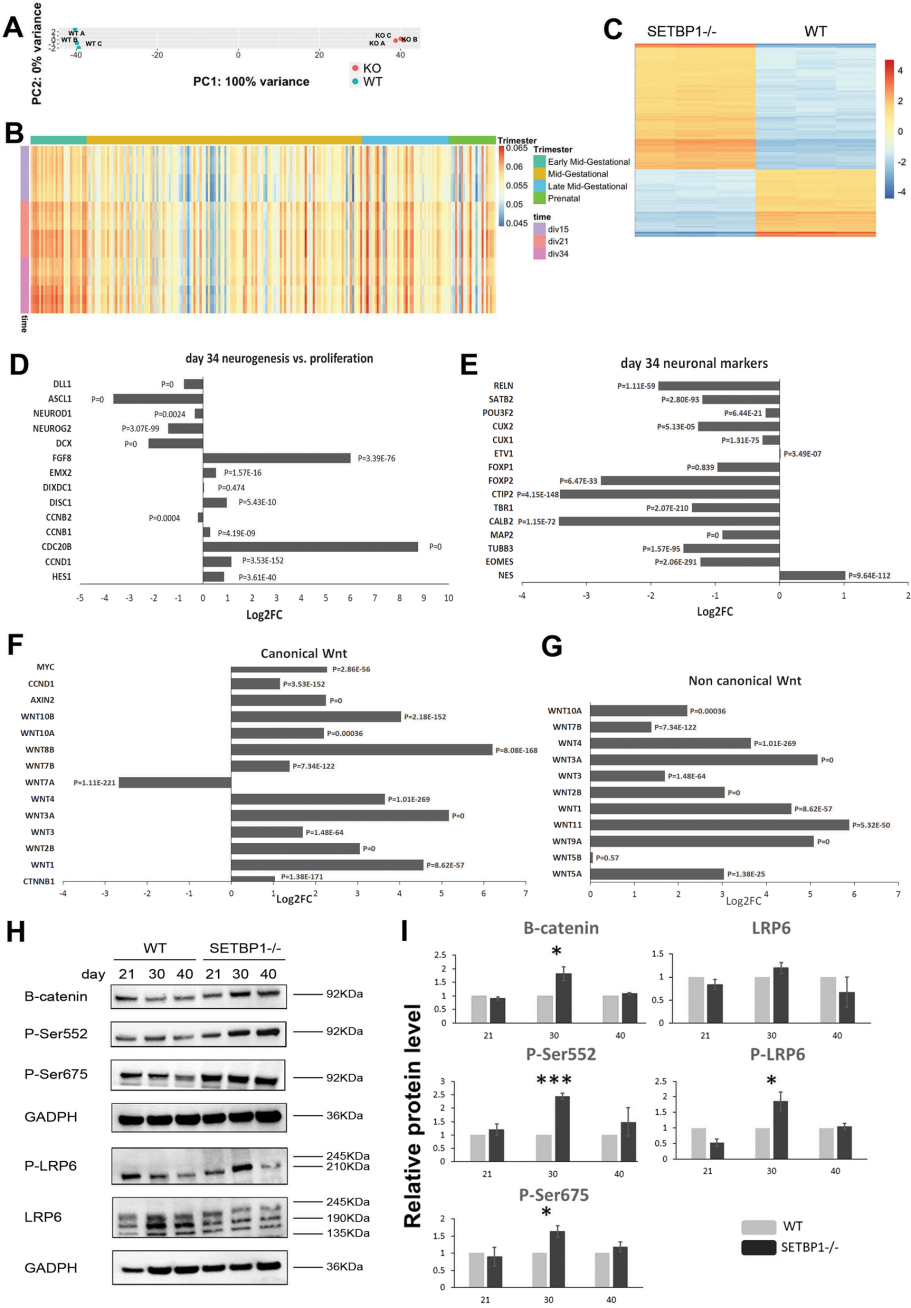
Genome-wide transcriptome profiling revealed SETBP1 regulation of Wnt signaling. **A** Principle component analysis (PCA) of the samples. **B** Heatmap depicting correlation with Allen Brain atlas at early, mid and late gestational trimesters and prenatal trimester. **C** Heatmap depicting 17,654 differentially expressed transcripts at day 34 (padj ≤ 0.1). **D** Example of neurogenic and proliferation related genes differentially expressed at day 34. **E** Example of neuronal marker genes downregulated at day 34, and upregulation of neuronal marker NES. **F, G** Differentially expressed genes associated with canonical non-canonical Wnt pathway at day 34. **H** Representative images of Western blot analysis for Wnt signaling proteins for WT and SETBP1-/- (Homo1). **I** Relative protein level of B-catenin, B-catenin p-S552 and p-S675, and LRP6 co-receptor and p-LRP6 at day 21, 30 and 40 to WT basal levels. Data from 3 independent differentiations analysed in duplicates or triplicates. Student’s T test was used to compare the expression between the two lines. (*p ≤ 0.05, **p ≤ 0.01, ***p ≤ 0.001). All tested proteins were normalized to GAPDH

To gain further insight into the molecular mechanisms underlying prolonged proliferation window of SETBP1-deficient NPCs, we performed a genome-wide transcriptome analysis of neural cells derived from the SETBP1-/- and isogenic WT control lines by RNAseq. To cover all stages of cellular abnormality, samples were collected from day 15 and day 21 (early and peak neural progenitor stage, respectively) and day 34, when abnormal NPC division and neurogenesis was becoming evident. Principle Component Analysis (PCA) showed that 100% of the variance is attributed to SETBP1 genotypes, while the biological replicates within SETBP1-/- or the control samples exhibit 0% variance statistically (Fig. 5A). Comparison of our hESCs-derived cortical gene expression to the BrainSpan Atlas of the Developing Human Brain (http://www.brainspan.org/), found the highest degree of correlation being at days 21 and 34 (Fig. 5B).

At a significant level of adjusted *P* ≤ 0.1, we identified 6060, 9997 and 17,654 differentially expressed transcripts at the three analysed time points, respectively (see example of day 34 in Fig. 5C). Consistent with the observed bias in NPC proliferation in the SETBP1-/- cultures, we observed an up-regulation of a number of neural proliferation marker genes such as *FGF8*, *DISC1*, *HES1, CDC20B* and *CCND1* at day 34 (Fig. 5D). This is complemented by a down-regulation of genes involved in neurogenesis such as *DLL1, ASCL1, NEUROD1, NEUROG2* and *DCX* (Fig. 5D). Moreover, at the same timepoint, basal progenitor (*TBR2*), pan neuronal (*TUBB3*, *MAP2*) and cortical layers specific marker genes (*TBR1*, *CTIP2*/*BCL11B*, *CUX1/2*, *SATB2, RELN*) were found down-regulated in the SETBP1-deficient cultures (Fig. 5E). In contrast, *NES* was twofold higher in the SETBP1-/- samples than the controls (Fig. 5E). These findings support the observed NPC SETBP1-deficient phenotype and demonstrate a further role for SETBP1 in cortical NPC proliferation and neurogenesis.

Using DAVID 6.8 gene functional classification tool [33] on the top 1000 differentially expressed protein coding genes, we identified that the top enriched gene ontology (GO) terms concerned mainly biological processes such as regulation of transcription, cell adhesion and extracellular matrix organization. KEGG (Kyoto Encyclopedia of Genes and Genomes, https://www.genome.jp/kegg) pathway analysis revealed Wnt, hippo, PI3K-Akt and ECM-receptor interaction signaling amongst the top enriched pathways up-regulated in SETBP1-/- cultures (Additional file 7: Fig. S6). All these pathways are highly relevant to the regulation of NPCs proliferation and neurogenesis [41–45].

Wnt signaling is known to play an important role in cortical development. Altered Wnt pathway was identified at all three differentiation stages, with the biggest changes observed at day 21 (FC 2.73, Padj = 0.0029) and day 34 (FC 2.53, Padj = 0.0014) (Additional file 7: Fig. S6B, C). We therefore examined further the gene set for transcripts involved in Wnt signaling (hsa04310) at day 34 (Fig. 5F, G and Additional file 8: Fig. S7A–C). Strikingly, the majority of the Wnt ligands, both canonical and non-canonical, were highly up-regulated in SETBP1-deficient cells, with fold change varying from 2.5 to 73 (Fig. 5F, G and Additional file 8: Fig. S7A, C). Also up-regulated were the canonical Wnt/*β*-catenin signaling responsive genes *C-MYC* (4.8×)*, CYCLIND1* (*CCND1,* 2x) and *AXIN2* (4.7×) (Fig. 5F and Additional file 8: Fig. S7A and C). In contrast, genes involved in *β*-catenin degradation complex (*GSK3* *β*, *CSNK*, *AXIN1/2*, *APC*) were mostly down-regulated (Additional file 6: Fig. S5A). We next used MAGMA (a tool for gene and gene-set analysis) to determine the association between risk variants identified from previously published ASD, and Intelligence GWAS, and enriched gene-sets identified from our RNAseq experiments. We chose to analyze these traits as they have clinical manifestations overlapping with SETBP1-HD. Amongst the analyzed gene-sets, “positive regulation of Wnt signaling” showed a nominal enrichment for genes associated with ASD in our day 34 dataset indicating a potential link between our in vitro model and the autistic traits observed in some of the SETBP1-HD patients (Additional file 9: Fig. S8A, B).

To ascertain that Wnt/*β*‐catenin signaling is indeed elevated in SETBP1-deficient NPCs at the protein level, we determined the level of *β*-catenin and Wnt co-receptor LRP6 in day 21, day 30 and day 40 neural cultures by Western blot (Fig. 5H). Activation of the canonical Wnt signaling results in N-terminal phosphorylation of *β*-catenin by GSK3*β*, leading to degradation of *β*-catenin [46, 47]. We found that the level of total *β*-catenin was significantly higher in SETBP1-/- cultures than the controls at day 30 (*P* = 0.031), although no differences were found at day 21 and 40 (Fig. 5I). It has been reported previously that C-terminal phosphorylation of *β*-catenin in serine 552 and serine 675 (p-S552 and p-S675) by AKT and PKA can enhance *β*-catenin/TCF reporter activation [48, 49]. We detected an average of 2.5-fold increase of p-S552 (*p* = 0.00024) and 1.5 fold increase of p-S675 (*p* = 0.019) in SETBP1-/- cultures than the controls at day 30 (Fig. 5I).

Another key phosphorylation event in the activation of the Wnt signaling cascade is the phosphorylation of the LRP5 and LRP6 co-receptors [50, 51], LRP6 is known to play a more dominant role during embryogenesis. We observed a near two-fold increase of phosphorylated LRP6 (p-LRP6) in day 30 SETBP1-/- NPCs than the isogenic control cells (*p* = 0.046, Fig. 5I). Together, these studies validated the increase of Wnt/*β*-catenin activation in SETBP1-deficient cells and provide the first demonstration of a regulatory role for SETBP1 in canonical WNT signaling in cortical NPCs.

### Pharmacological inhibition of Wnt/β-catenin pathway rescues proliferation defect of SETBP1-/- cortical NPCs and ameliorates MGE fate induction deficit

**Fig. 6:**
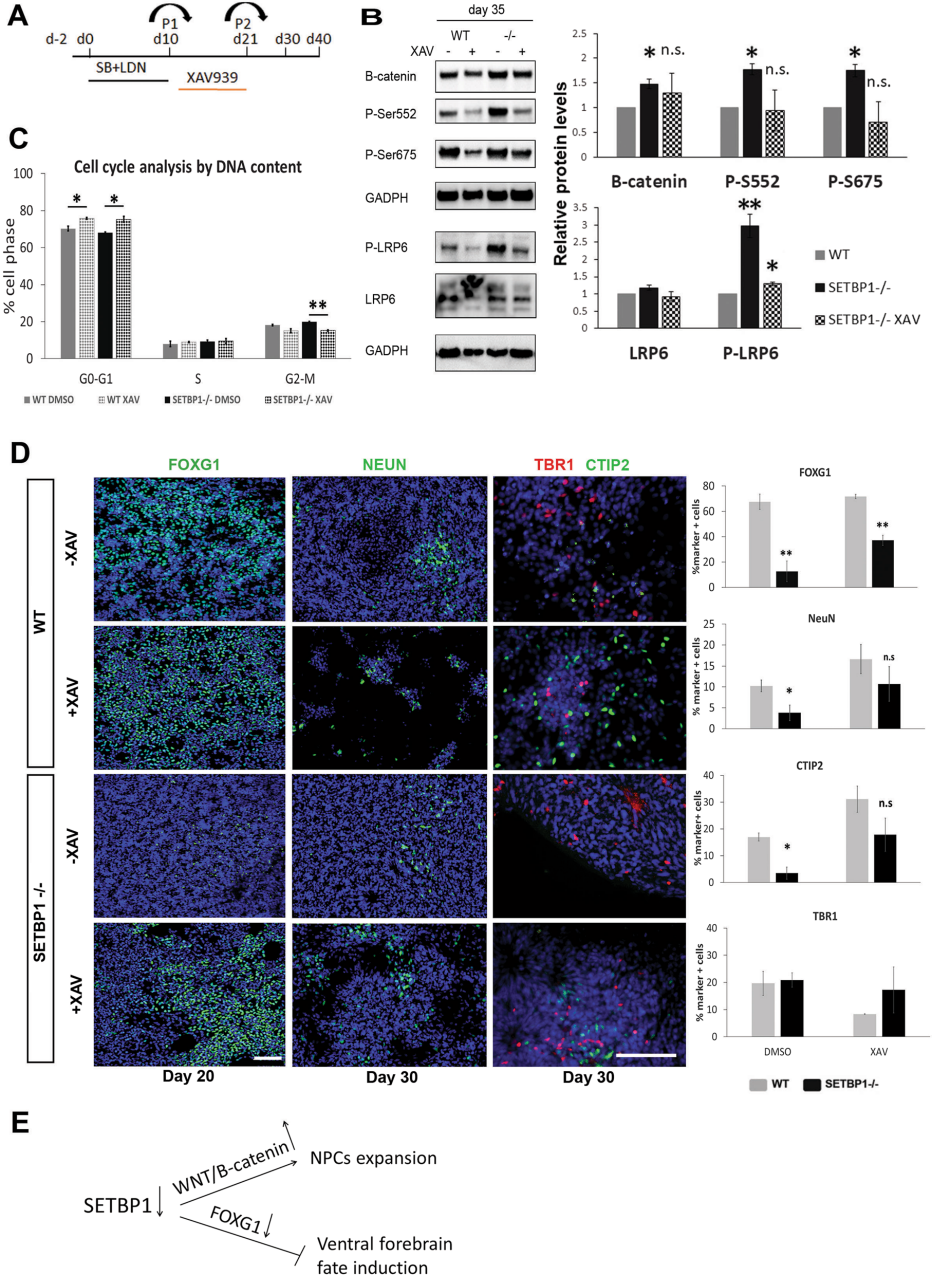
Phenotypic rescue of SETBP1 deficiency by pharmacological interrogation of Wnt signaling. **A** Experimental scheme. Differentiation cultures under basal condition (control) or exposed to 2uM XAV939 from day 11 to day 21. **B** Western blot analysis at day 35 for the effects of XAV treatment on WNT signaling proteins. Data was obtained from 2 independent differentiations analysed in duplicates or triplicates and shown as relative levels to the WT. Student’s *T* test was used to compare the expression between the two lines, B-catenin basal *P* = 0.033, XAV *P* = 0.542, S552 basal *P* = 0.023, XAV *P* = 0.906, S674 basal *P* = 0.024, XAV *P* = 0.554, LRP6 basal *P* = 0.153, XAV *P* = 0.644, P-LRP6 basal *P* = 0.004, XAV *P* = 0.012. **C** Effect of XAV treatment on cell cycle profile at day 35. Data presented as mean ± s.e.m of 2 independent experiments in triplicates. Student’s *T* test, one tail, was used to compare the expression between the two lines and the two conditions (WT basal vs. XAV G0-G1 *P* = 0.029, S *P* ≥ 0.05, G2-M *P* ≥ 0.05, SETBP1-/- basal vs. XAV G0-G1 *P* = 0.021, S *P* ≥ 0.05, G2-M *P* = 0.0045. **D** Immunofluorescence images of cultures in basal (DMSO) or XAV treated conditions at day 20 and 30. Cell nuclei were labelled by DAPI. Scale bar: 100uM. Bar- graphs showing quantification of FOXG1 (green) positive NPS at day 20 and CTIP2 (green), TBR1 (red) and NeuN (green) positive neurons at day 30. Student’s *T* test was used to compare the expression between the two lines, FOXG1^+^ cells: basal *P* = 0.002, XAV *P* = 0.001; CTIP2^+^ cells: basal *P* = 0.029, XAV *P* = 0.214; TBR1^+^ cells: basal *P* = 0.672 XAV *P* = 0.258, NeuN^+^ cells: basal *P* = 0.038, XAV *P* = 0.333 (*p ≤ 0.05, **p ≤ 0.01). **E** Schematic illustration depicting the role of SETBP1 in regulating NPC pool expansion and forebrain fate induction

To establish a causal relationship between the increased Wnt/*β*-catenin signaling and over proliferation of SETBP1-deficient NPCs, we interrogated Wnt signaling using XAV939 (XAV), a small molecule tankyrase inhibitor that stabilizes Axin and stimulates*β*-catenin degradation [52]. SETBP1-/- and WT NPC cultures were exposed to XAV for 10 days from day 11, a time window prior to the phenotypic manifestation (Fig. 6A and Additional file 10: Fig. S9A). Wnt signaling inhibition by XAV was verified by evident reduction in total *β*-catenin, p-S552/p-S675 as well as p-LRP6 comparing treated SETBP1-/- with respective to the no XAV sister cultures in both WT and SETBP1-/- cultures (Fig. 6B). Importantly, after XAV treatment, total *β*-catenin, p-S552 and p-S675 in SETBP1-/- cells were no longer different to the isogenic control cells without XAV treatment. As a control for inhibitor specificity, the levels of the GAPDH were not affected by XAV treatment.

The effect on XAV treatment in NPCs proliferation was examined via cell cycle analysis of DNA content (Fig. 6C). An increase of cells in G1 phase and a decrease of cells in G2-M was observed for both SETBP1-/- and control NES^+^ NPCs. In XAV treated SETBP1-/- cultures, the number of cells in G2-M phase was restored to a level similar to those in the WT cultures with or without XAV (15.18 ± 0.51 and 15.02 ± 1.07, *P* = 0.902). We next examined the effect of XAV on the cultures at day 20, 30, 40 and 50 (Fig. 6D and Additional file 10: Fig. S9). Compared to non-treated SETBP1-/- cultures, immunostaining revealed that, at day 20 there is *a* > 50% increase in FOXG1^+^ NPCs in the SETBP1-/- cultures (12.62 ± 8.22 vs. 37.15 ± 4.09, *P* = 0.027). (Fig. 6D). Moreover, as expected, XAV treatment in the WT cultures accelerated neuronal differentiation as demonstrated by a reduced number of PAX6^+^ and NES^+^ cells and concurrent increase of NeuN^+^ and MAP2^+^ cells in comparison to no XAV sister control cultures (Additional file 10: Fig. S9B, C). XAV treatment also resulted in a significant increase in CTIP2^+^ cells in both WT and SETBP1-/- cultures, so the CTIP2^+^ cell numbers in the treated SETBP1-/- cultures reached to a similar levels to those in the basal WT cultures (eg. day 30 WT untreated 16.97 ± 1.50 vs. SETBP1-/- XAV 17.82 ± 6.20, *P* = 0.844) (Fig. 6D and Additional file 10: Fig. S9B, C and E). XAV treatment didn’t have a significant effect on the number of TBR1^+^ and SATB2^+^ cell populations on all time points although a decrease of TBR1^+^ cells was detected at day 50 (Fig. 6D and Additional file 10: Fig. S9). Compared to non-treated SETBP1-/- cultures, cells in XAV treated cultures exhibited pronounced neuronal arborisation similarly to those in the WT control cultures without XAV (Additional file 10: Fig. S9B).

WNT inhibition via XAV at day 0–10 was part of the MGE differentiation paradigm (Fig. 4E). Since the production of NKX2.1^+^ progenitors was lower in SETBP1-deficient cultures, we investigated a potential effect of extended XAV treatment (d0-20) on MGE differentiation of SETBP1 mutant cells. We found that longer XAV exposure resulted in an increase of NKX2.1^+^ cells in both the WT and SETBP1 mutant cultures compare to the standard MGE protocol. Under XAV d0-20 condition, while NKX2.1^+^ cell numbers still appear lower in the SETBP1 mutant cultures compared to the WT controls, no statistical differences were reached (Additional file 11: Fig. S10). Together, our data demonstrates that inhibition of Wnt/*β*-catenin signaling can restore the neurogenesis and fate induction defects of SETBP1-/- NPCs and thus provide a functional verification that SETBP1 is playing a role in WNT signaling.

## Discussion

During development, corticogenesis is tightly regulated temporally and spatially with a progressive temporal restriction in progenitor potential to ensure the generation of correct numbers of neurons in time and space. NPCs firstly reproduce themselves to expand the progenitor pool via symmetric or proliferative cell divisions [53, 54]. Later, cell division pattern changes to asymmetric neurogenic divisions that generates an NPC that re-enters the cell cycle and a postmitotic neuron, or symmetric neurogenic divisions that yield two neurons [55, 56]. Defects in this process can lead to a wide range of brain malformations such as micro- or macrocephaly. Using genome-edited hESC and in vitro cortical differentiation as an experimental model, we report here that loss of *SETBP1* impairs neurogenesis and affects neural progenitor fate choice, thus identifying *SETBP1* as an important regulator governing the delicate balance between NPC expansion and terminal differentiation. This newly discovered biological function of *SETBP1* in human neural development is consistent with its high-level expression in the developing cortex and its evolutionary conservation.

Dysregulation of Wnt signaling in *SETBP1*-deficient neuronal cultures presents another interesting new finding of this study. This regulatory relationship was demonstrated at both transcript and protein level with further support of functional interrogation and phenotypic rescue. Wnt signaling is known to play an important role in cortical development. Elevated canonical Wnt signaling by enforced expression of stabilized *β*-catenin promotes cell cycle re-entry of NPCs, leading to their excessive expansion in telencephalon [57]. A prominent feature of our SETBP1-deficiency model is prolonged NPC proliferation, due to shortened cell-cycle length and reduced cell cycle exit rate. Therefore, the increased Wnt signaling in SETBP1-/- cultures is likely a key contributor to the aberrant NPC proliferation. Schinzel–Giedion syndrome-associated SETBP1 mutations have recently been shown to impair mouse neurogenesis and proliferation of iPSC-derived NPCs although via Wnt independent mechanisms [57, 58].

Interestingly, dysregulated Wnt signaling have been recently reported in a growing number of studies employing patient-derived iPSC or CRISPR/Cas9 edited hESC models of neurodevelopmental disorders that include schizophrenia, ASD and intellectual disability [58–62]. Elevated Wnt signaling has been implicated as a cause of the macrocephaly observed in ASD patients [63]. Despite the relatively small sample size of the ASD GWAS, we observe a tendency for enrichment of common variants associated with ASD amongst Wnt signaling genes. The current study suggests that increased Wnt signaling may be also an underlying mechanism of the cognitive and motor impairment observed in patients with SETBP1 disorder.

In murine myeloid progenitor cells, SETBP1 has been suggested to exert transcription factor function through binding of Hoxa9/10 promoters [64], while binding of Setbp1 to Runx1 promoter in a similar cell model caused a downregulation of RUNX1 expression [65]. Moreover, SETBP1 can bind to gDNA in AT-rich promoter regions and trigger gene expression via recruiting HCF1/KMT2A/PHF8 epigenetic complex [66]. Another way suggested by which SETBP1 exert its regulatory function is via interaction with oncogene SET [67]. Most of these studies were carried in myeloid progenitor cells or SGS mutation related models resembling SETBP1 gain of function and a direct regulation of Wnt signaling by SETBP1 in the context of human brain development has not been reported to date. While further investigation is needed to unravel how SETBP1 modulate Wnt/*β*-catenin signaling in neural cells, our work uncover a new function of SETBP1 and a therapeutic road to explore in the context of SETBP1 haploinsufficiency.

*FOXG1* is the most significantly down-regulated transcript in SETBP1-/- NPC cultures. Given the established role for Foxg1/FOXG1 in ventral forebrain development, its reduction is the likely cause of compromised generation of LGE- and MGE-like NPCs in the SETBP1-/- cultures [38–40]. Foxg1 has been shown to directly suppress Wnt ligand expression and Wnt signaling in model organisms [68, 69]. Therefore, reduced FOXG1 may also attribute to the elevated WNT activity in SETBP1-deficient cultures. The MGE differentiation protocol used in this study applies WNT inhibition by exposing the cultures to a Wnt inhibitor XAV at day 0–10 to facilitate ventral patterning. We observed a decreased production of NKX2.1^+^ MGE like progenitors, a phenotype not appeared to be associated with a change of cell cycle characteristics. However, extended XAV exposure (day 0–20) was able to partially reverse the number of NKX2.1^+^ cells. This observation implies WNT overactivation as a likely underling cause of the SETBP1-dependent ventral fate deficit. It is interesting to note that, unlike the cortical NPC neurogenesis deficit being mostly confined to the SETBP1-/- genotype, compromised ventral fate induction was also evident in the SETBP1+/- cultures, thus this neurodevelopmental phenotype might be more relevant to pathogenesis of SETBP1-HD.

Moreover, SETBP1 deficiency resulted in a significant reduction of CTIP2^+^ cells and slightly higher numbers of TBR1^+^ cells albeit not statistically significant. Previous studies in mouse and zebrafish suggest that Foxg1 confers the competence of cortical NPCs for the characteristic ordered generation of layer-specific neuronal subtypes by coordinating Wnt and Shh signaling pathways in the telencephalon [70, 71]. Therefore, SETBP1-dependent FOXG1 hypofunction may also contribute to the distorted neuronal production in our SETBP1-/- cultures. Our data opens new avenues to explore the functional link between FOXG1 and SETBP1 genes in the context of brain development.

### Limitations

Despite the robust findings made in independent isogenic PSC lines, the distorted NPC proliferation and neurogenic differentiation were more evident in cultures with homozygous deletion of SETBP1 while the SETBP1+/- cultures exhibit either mild or no deficits. However, SETBP1-HD arose from heterozygous gene deletion of SETBP1 and it is possible that children suffering from this disorder may harbour other genetic variants or there exist a specific modifier gene that aggravate the loss of SETBP1. Future work on patient-iPSC derived neural cultures is thus needed, albeit the challenge in collecting tissue samples for rare disorders such as this.

### Conclusions

In conclusion, we identified an important role for SETBP1 in controlling forebrain progenitor expansion and neurogenic differentiation. Disturbed NPC proliferation and neuronal differentiation can lead to brain malformation as it happens in other neurodevelopmental disorders. Our finding of disturbed cortical progenitor proliferation and defective neurogenesis in SETBP1-/- model lend itself an invaluable tool to further investigate the aetiology of SETBP1 disorder. Moreover, this study revealed a novel regulatory link between SETBP1 and Wnt/*β*-catenin signaling during human cortical neurogenesis and provides mechanistic insights into structural abnormalities and potential therapeutic avenues for SETBP1 disorder.

## Supplementary Information

**Additional file 1: Table S1. **Primers for PCR and qPCR used in the study. qPCR primers were designed to anneal at 60C.

**Additional file 2: Fig. S1.** Generation of the SETBP1-deficient hESC lines. **A** Schematic illustration of the targeted allele of SETBP1 locus. The 5’ and 3’ homologous arms (HA) corresponding to exon 4 and part of intron 4/5 are indicated in yellow, which are flanked by a PGKpuropA selection cassette in the targeting vector. The positions of the two nested PCR primer pairs for screening homologous recombination (HR) at the 5’ and 3’ are indicated in black and grey arrows, respectively. **B** Agarose gels showing the predicted PCR amplicon from the targeted clones using the 5’ and 3’ primer pairs, respectively (lanes marked with *). **C** Alignment of Sanger sequencing product the sequence flanking gRNA1 target locus for the cell lines used in the study. A deletion of 5bp (CAGCG) was detected in the second allele of the SETBP1-/- lines following the second round of CRISPR/Cas9 editing. **D** Relative quantification of SETBP1 mRNA levels with primers binding downstream of the region targeted by the gRNAs (Forward in exon 4-5 junction and reverse in exon 5-6 junction) Student’s T test was used to compare the expression levels between the mutant clones and the parental (WT) line. WT vs. Homo1 *P*=0.003, WT vs. Homo2 P=0.0005 (**p ≤ 0.01, **p ≤ 0.001).

**Additional file 3: Fig. S2. **Validation of cortical induction by qPCR. **A** qPCR analysis of pluripotency and telencephalic marker genes. RNA samples were harvested every 5 days from day 0 to 30. Levels of mRNA expression were normalized to day 0 mRNA levels. SETBP1-/- levels were normalized against WT levels. Data shown are Log2RQ levels. **B** qPCR analysis of neural stem cell and neuronal marker genes. RNA samples were harvested every 5 days from day 21 to 50. Levels of mRNA expression were normalized to day 21. SETBP1-/- levels were normalized against WT levels. Data shown are RQ levels. Data shown as mean ± s.e.m of two independent differentiations analyzed in triplicates. Student’s T test was used to compare the expression between the two lines (*p ≤ 0.05, **p ≤ 0.01, ***p ≤ 0.001).

**Additional file 4: Fig. S3.** Reduced neuronal production in SETBP1-/- cultures. **A** The WT control, SETBP1+/- and SETBP1-/- cultures were immunostained for NeuN at days 30, 40 and 50 and MAP2 and NESTIN at day 50. **B** Graphs showing quantitative measurements for NeuN. Data presented as mean ± s.e.m for each genotype with a minimum of two independent experiments carried out per line (WT = 5, HET1=2, HET2 = 2, Homo1 = 3, and Homo2 = 2). One-way ANOVA test, Bonferroni Post Hoc; *p≤0.05, Scale bar: 100uM.

**Additional file 5: Fig. S4.** Flow cytometry gating strategy for Cell cycle analysis based on DAPI content. **A** P1 snapshot, **B** P2 or single cell population snapshot, **C** Nestin+ progenitors population, **D** Cell cycle phases profile in WT cultures Nestin+ progenitors, **E** Cell cycle phases profile in SETBP1-/- cultures Nestin+ progenitors.

**Additional file 6: Fig. S5.** Proliferation analysis on day 35 MGE cultures. Quantification of EdU and Ki67 positive cells in ventral differentiation at day 35. Data presented as mean ± s.e.m. for each genotype with three biological replicas carried out per line (WT, HET1, HET2, Homo1, and Homo2) ANOVA test, EdU *P* = 0.557, Ki67. *P* = 0.657.

**Additional file 7: Fig. S6.** Differentially expressed KEGG pathways. **A** Top 20 enriched KEGG pathways in day 15 dataset. **B** Top 20 enriched KEGG pathways in day 21 dataset. **C** Top 20 enriched KEGG pathways in day 34 dataset. Differentially expressed genes used in this analysis were restricted to those with adjusted *p* value<0.1 and a FC>1.5. Benjamini-Hochberg correction was applied for multiple comparisons.

**Additional file 8: Fig. S7.** Altered expression of genes associated with Wnt pathway at day 34. **A** Transcriptomic expression of canonical-Wnt related genes represented as Log2FC. **B** Heatmap depicting differentially expressed transcripts for Wnt signalling (GO:0016055). Differentially regulated transcripts used in this analysis were restricted to those with adjusted *p* value<0.1, and a FC>1.5. Benjamini-Hochberg correction was applied for multiple comparisons. **C** WNT signalling pathway graphical representation rendered by Pathview. Image shows up and downregulated pathway components in the SETBP1-/- compared with parental for canonical, planar cell polarity and calcium pathways after intersecting the data with KEGG pathway maps.

**Additional file 9: Fig. S8**. MAGMA enrichment results. **A** Barplots indicating enrichment for common variants associated with ASD at day 21 (left) and day 34 (right). **B** Barplots indicating enrichment for common variants associated with Intelligence at day 21 (left) and day 34 (right). Green dashed line indicates nominal *p* value threshold, red dashed line indicates Bonferroni adjusted-*p*-value threshold for multiple comparisons.

**Additional file10: Fig. S9.** Phenotype recovery after XAV939 treatment. **A** Experimental scheme indicating window for XAV939 treatment. **B**, **C** Phase contrast and fluorescent images of cultures in basal and XAV condition at day 40 and day 50. The blue staining in the 3rd and 5th column are nuclei stained with DAPI. Scale bar: 100uM. **D**–**F** Bar- graphs showing quantification of TBR1, CTIP2 and SATB2 positive neurons from basal and XAV939 treated cultures at day 40 and 50. Data presented as mean ± s.e.m for each genotype with a minimum of three independent experiments carried out per line. Student’s T test was used to compare the expression between the two lines in each condition, Basal cultures: Day 40 TBR1+ cells *P*=0.714, CTIP2+ cells *P*=0.03, SATB2+ cells *P*=0.476; Day 50 TBR1+ cells *P*=0.390, CTIP2+ cells *P*=0.035, SATB2+ cells *P*=0.166. XAV treated cultures: Day 40 TBR1+ cells *P*=0.177, CTIP2+ cells *P*=0.979, SATB2+ cells *P*=0.255; Day 50 TBR1+ cells *P*=0.016, CTIP2+ cells *P*=0.830, SATB2+ cells *P*=0.298. (*p ≤ 0.05).

**Additional file11: Fig. S10.** Effect of extended XAV939 treatment in ventral cultures. **A**, **B** Experimental scheme indicating windows for XAV939 treatment in the standard MGE protocol and XAV extended protocol. **C** Immunostaining of day 20 cultures for NKX2.1 (green) and COUP-TFII (red). **D** Quantitative data of NKX2.1 marker expression presented as mean ± s.e.m. for each genotype with three biological replicas carried out per line (WT, HET1, HET2, Homo1, and Homo2) **E** Expression of PAX6 (red). **F** Expression of GSH2 (red). Dapi was used to label all nuclei. Scale bar: 100uM.

## Data Availability

The raw RNA sequencing data generated during the current study is available in the GEO repository, accession number GSE180185. The datasets used and/or analysed during the current study are available from the GitHub repository (https://github.com/DanCF93/Cardo-et-al-2021) and the corresponding author on reasonable request. All data generated or analysed during this study are included in this published article [and its supplementary information files].

## Abbreviations

ASD: Autism spectrum disorder
CRISPR: Clustered regularly interspaced palindromic repeats
CAS9: CRISPR-associated protein 9
HESC: Human embryonic stem cell
iPSC: Induced pluripotent stem cell
LGE: Lateral ganglionic eminence
MGE: Medial ganglionic eminence
N-Cad: N-cadherin
NES: Nestin
NPC: Neural progenitor cell
KEGG: Kyoto Encyclopedia of genes and genomes
PCA: Principle component analysis
SET: SET nuclear proto-oncogene
SETBP1: SET binding protein 1
SETBP1 ±: SETBP1 heterozygous deletion
SETBP1-/-: SETBP1 homozygous deletion
SETBP1-HD: SETBP1 haploinsufficiency disorder
SGS: Schinzel–Giedion syndrome
WNT: Wingless and Int-1
WT: Wild type
