# Supplementary material for: Impaired neurogenesis and neural progenitor fate choice in a human stem cell model of SETBP1 disorder
Source: Mol Autism. 2023 Feb 20;14:8. doi: 10.1186/s13229-023-00540-x (PMC9940404; doi:10.1186/s13229-023-00540-x)
Supplement: Supplementary file 1 — Additional file 1: Table S1. Primers for PCR and qPCR used in the study. qPCR primers were designed to anneal at 60C. [file 13229_2023_540_MOESM1_ESM.docx]

**Supplementary Table 1. Primers for PCR and qPCR used in the study. qPCR primers were designed to anneal at 60C.**

| **Amplicon/**  **gene** | **Forward 5’-3’** | **Reverse 5’-3’** | **Size**  **(bp)** |
| --- | --- | --- | --- |
| SETBP1 5’HA | TAAGCGAATTCAAGGCCCAGGGTAAGAAAGG | CGAATGTCGAC CCACCACCGCTTTAATGGAC | 502 |
| SETBP1 3’HA | GTACAGCGGCCGCGGGCTTTGCACCTCAGACAC | ACATGGGATCCTTTAGGGACCTGCCCAGGAC | 551 |
| NHEJ gRNA1 | GCTGAGTAGCGCAGACAAA | CTTTTTCCTTGGCCTGTGTC | 180 |
| NHEJ gRNA2 | CCGGCAAAAGCATCTCATTG | TGGCACCGGGTAATAGTGAT | 240 |
| 5’HA Nested 1 | CAGAATTGCTTCATCAGTCCAG | GCTCGTAGAAGGGGAGGTTG | 1713 |
| 5’HA Nested 2 | CTGGCAGAGAAACTGCAAGC | GGCTTGTACTCGGTCATGGTA | 1157 |
| 3’HA Nested 1 | AGCTGCAAGAACTCTTCCTCAC | GACACAGTTATAAGGTCTGCTGG | 1531 |
| 3’HA Nested 2 | CGATGATCTAGAGCTCGCTGA | CTCCTTCCACTTAGTGCACG | 944 |
| OCT4 | CGACCATCTGCCGCTTTGAG | CCCCCTGTCCCCCATTCCTA | 573 |
| NANOG | AGCCTCTACTCTTCCTACCACC | TCCAAAGCAGCCTCCAAGTC | 278 |
| FOXG1 | TGGCCCATGTCGCCCTTCCT | GCCGACGTGGTGCCGTTGTA | 77 |
| GLI3 | GCTCCACGACCACTGAAAAG | CTGTCCAGGACTTTCATCCTCATTA | 125 |
| OTX2 | TGCCAAAAAGAAGACATCTCCA | AAGCTGGGCTCCAGATAGACAC | 137 |
| PAX6 | AGAGAATACCAACTCCATC | GATAATGGGTTCTCTCAAG | 152 |
| NES | AGCAGGAGAAACAGGGCCTAC | CTCTGGGGTCCTAGGGAATTG | 218 |
| SOX1 | GGAAGGTCATGTCCGAGGCC | ACTTGTCCTTCTTGAGCAGCG | 138 |
| SOX2 | CATGGCAATCAAAATGTCCA | TTTCACGTTTGCAACTGTCC | 102 |
| HES1 | GTGTCAACACGACACCGGAT | GGAATGCCGCGAGCTATCTT | 161 |
| MASH1 | GTCCTGTCGCCCACCATCTC | CCCTCCCAACGCCACTGAC | 251 |
| TBR1 | GACTCAGTTCATCGCCGTCA | GCCGGTGTAGATCGTGTCAT | 149 |
| CTIP2 | CTCCGAGCTCAGGAAAGTGTC | TCATCTTTACCTGCAATGTTCTCC | 129 |
| GAPDH | ATGACATCAAGAAGGTGGTG | CATACCAGGAAATGAGCTTG | 177 |
| SETBP1 | CATGTCTCCAGGGATGCCAAG | GAGCCGACTGCATCCGAC | 189 |
