# Supplementary figures and images for: Impaired neurogenesis and neural progenitor fate choice in a human stem cell model of SETBP1 disorder
Source: Mol Autism. 2023 Feb 20;14:8. doi: 10.1186/s13229-023-00540-x (PMC9940404; doi:10.1186/s13229-023-00540-x)

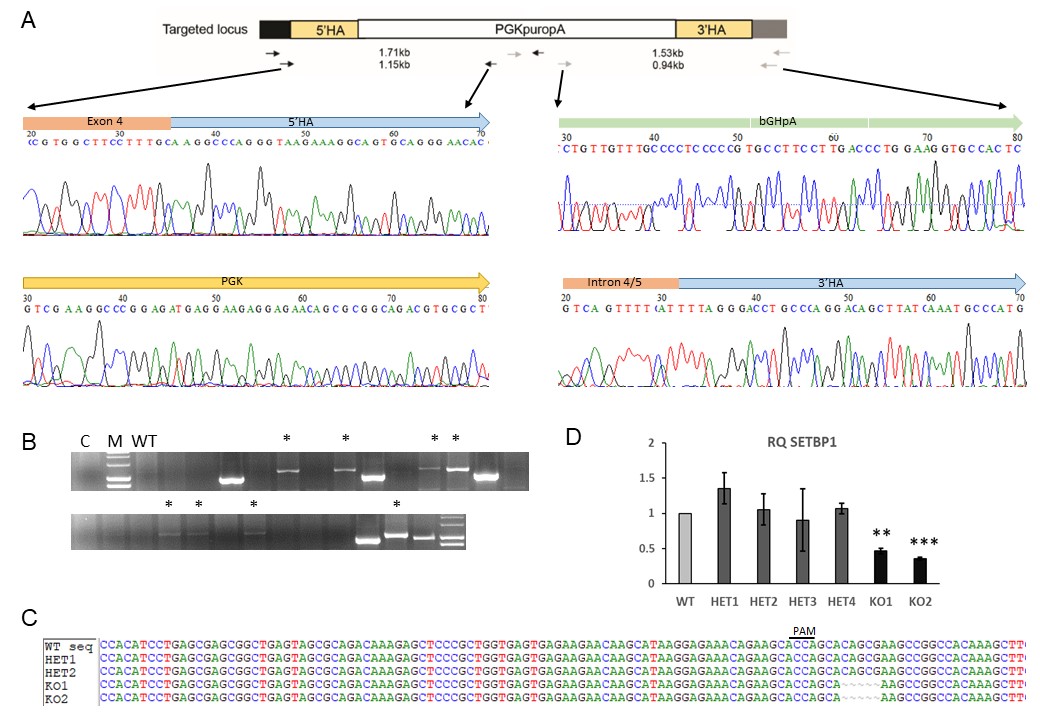

Supplement: Supplementary file 2 — Additional file 2: Fig. S1. Generation of the SETBP1-deficient hESC lines. A Schematic illustration of the targeted allele of SETBP1 locus. The 5’ and 3’ homologous arms (HA) corresponding to exon 4 and part of intron 4/5 are indicated in yellow, which are flanked by a PGKpuropA selection cassette in the targeting vector. The positions of the two nested PCR primer pairs for screening homologous recombination (HR) at the 5’ and 3’ are indicated in black and grey arrows, respectively. B Agarose gels showing the predicted PCR amplicon from the targeted clones using the 5’ and 3’ primer pairs, respectively (lanes marked with *). C Alignment of Sanger sequencing product the sequence flanking gRNA1 target locus for the cell lines used in the study. A deletion of 5bp (CAGCG) was detected in the second allele of the SETBP1-/- lines following the second round of CRISPR/Cas9 editing. D Relative quantification of SETBP1 mRNA levels with primers binding downstream of the region targeted by the gRNAs (Forward in exon 4-5 junction and reverse in exon 5-6 junction) Student’s T test was used to compare the expression levels between the mutant clones and the parental (WT) line. WT vs. Homo1 P=0.003, WT vs. Homo2 P=0.0005 (**p ≤ 0.01, **p ≤ 0.001). [file 13229_2023_540_MOESM2_ESM.jpg]

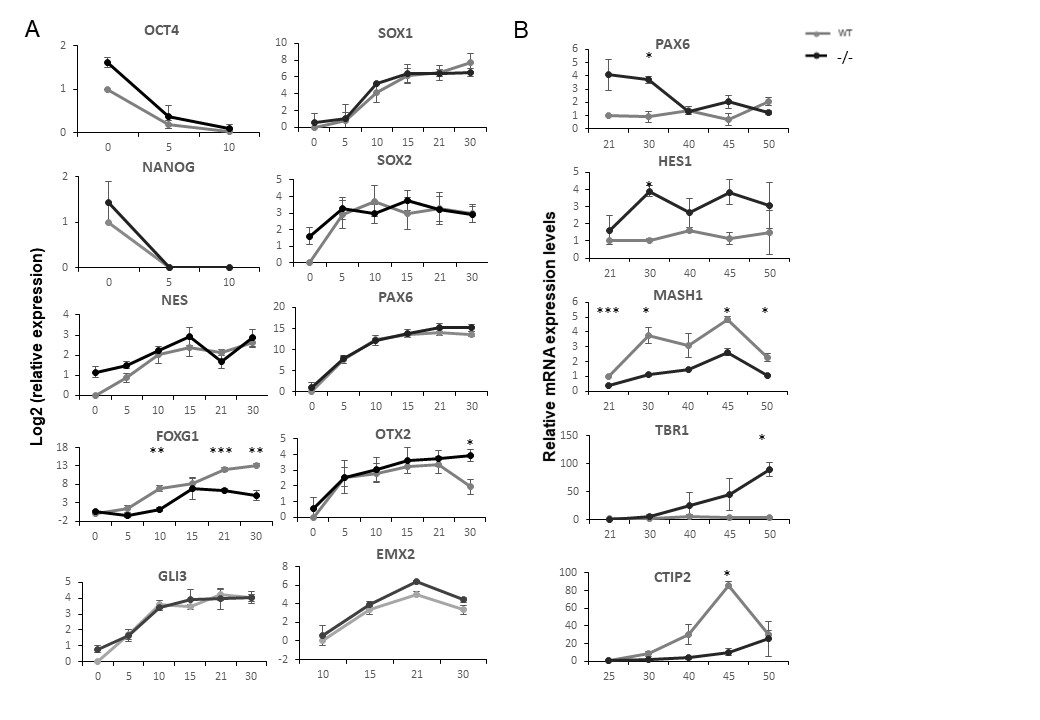

Supplement: Supplementary file 3 — Additional file 3: Fig. S2. Validation of cortical induction by qPCR. A qPCR analysis of pluripotency and telencephalic marker genes. RNA samples were harvested every 5 days from day 0 to 30. Levels of mRNA expression were normalized to day 0 mRNA levels. SETBP1-/- levels were normalized against WT levels. Data shown are Log2RQ levels. B qPCR analysis of neural stem cell and neuronal marker genes. RNA samples were harvested every 5 days from day 21 to 50. Levels of mRNA expression were normalized to day 21. SETBP1-/- levels were normalized against WT levels. Data shown are RQ levels. Data shown as mean ± s.e.m of two independent differentiations analyzed in triplicates. Student’s T test was used to compare the expression between the two lines (*p ≤ 0.05, **p ≤ 0.01, ***p ≤ 0.001). [file 13229_2023_540_MOESM3_ESM.jpg]

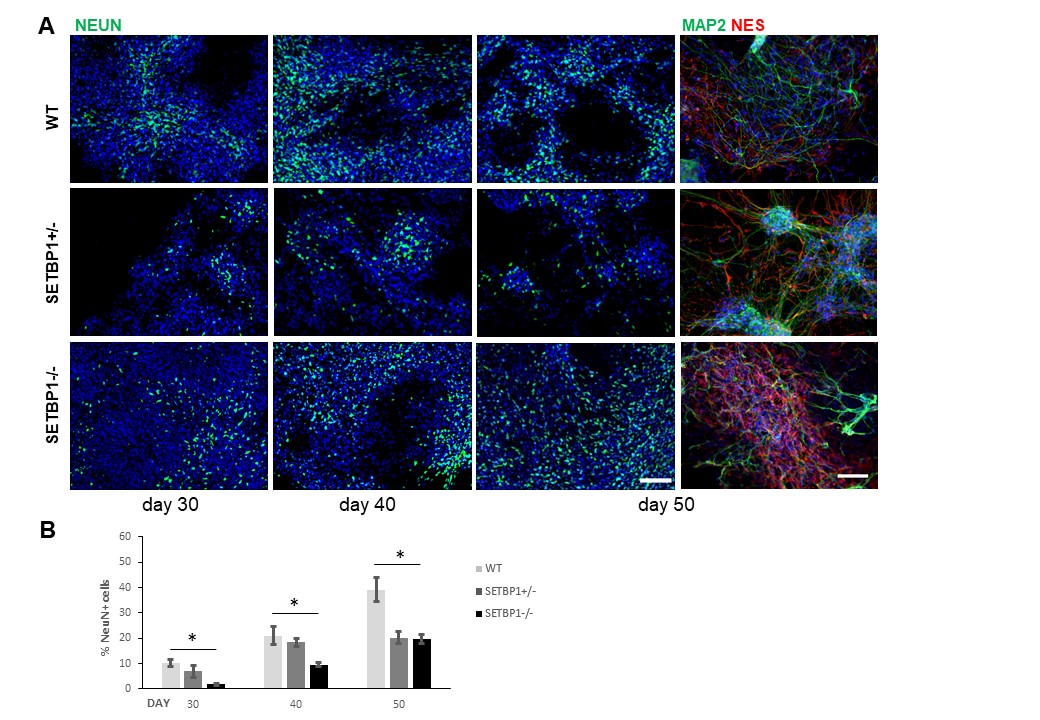

Supplement: Supplementary file 4 — Additional file 4: Fig. S3. Reduced neuronal production in SETBP1-/- cultures. A The WT control, SETBP1+/- and SETBP1-/- cultures were immunostained for NeuN at days 30, 40 and 50 and MAP2 and NESTIN at day 50. B Graphs showing quantitative measurements for NeuN. Data presented as mean ± s.e.m for each genotype with a minimum of two independent experiments carried out per line (WT = 5, HET1=2, HET2 = 2, Homo1 = 3, and Homo2 = 2). One-way ANOVA test, Bonferroni Post Hoc; *p≤0.05, Scale bar: 100uM. [file 13229_2023_540_MOESM4_ESM.jpg]

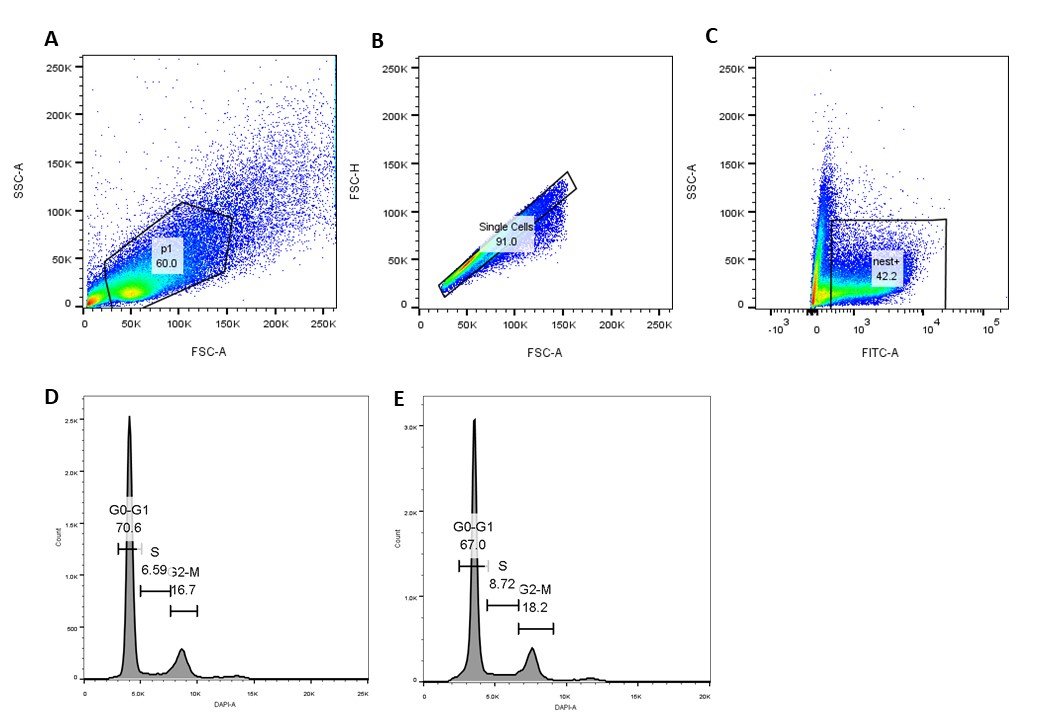

Supplement: Supplementary file 5 — Additional file 5: Fig. S4. Flow cytometry gating strategy for Cell cycle analysis based on DAPI content. A P1 snapshot, B P2 or single cell population snapshot, C Nestin+ progenitors population, D Cell cycle phases profile in WT cultures Nestin+ progenitors, E Cell cycle phases profile in SETBP1-/- cultures Nestin+ progenitors. [file 13229_2023_540_MOESM5_ESM.jpg]

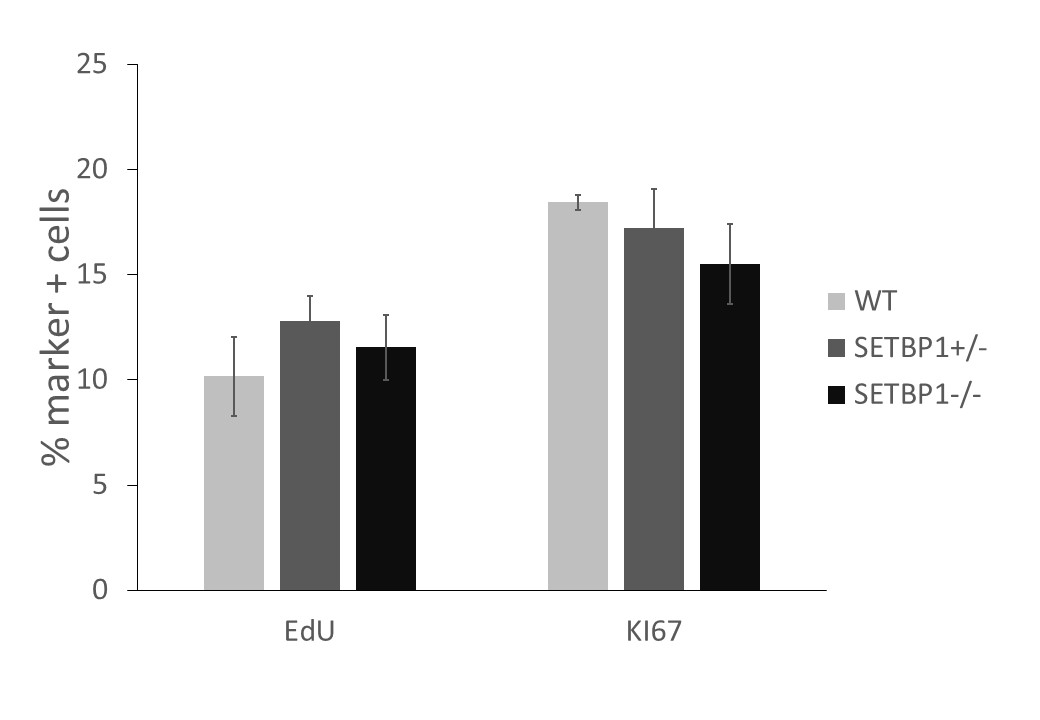

Supplement: Supplementary file 6 — Additional file 6: Fig. S5. Proliferation analysis on day 35 MGE cultures. Quantification of EdU and Ki67 positive cells in ventral differentiation at day 35. Data presented as mean ± s.e.m. for each genotype with three biological replicas carried out per line (WT, HET1, HET2, Homo1, and Homo2) ANOVA test, EdU P = 0.557, Ki67. P = 0.657. [file 13229_2023_540_MOESM6_ESM.jpg]

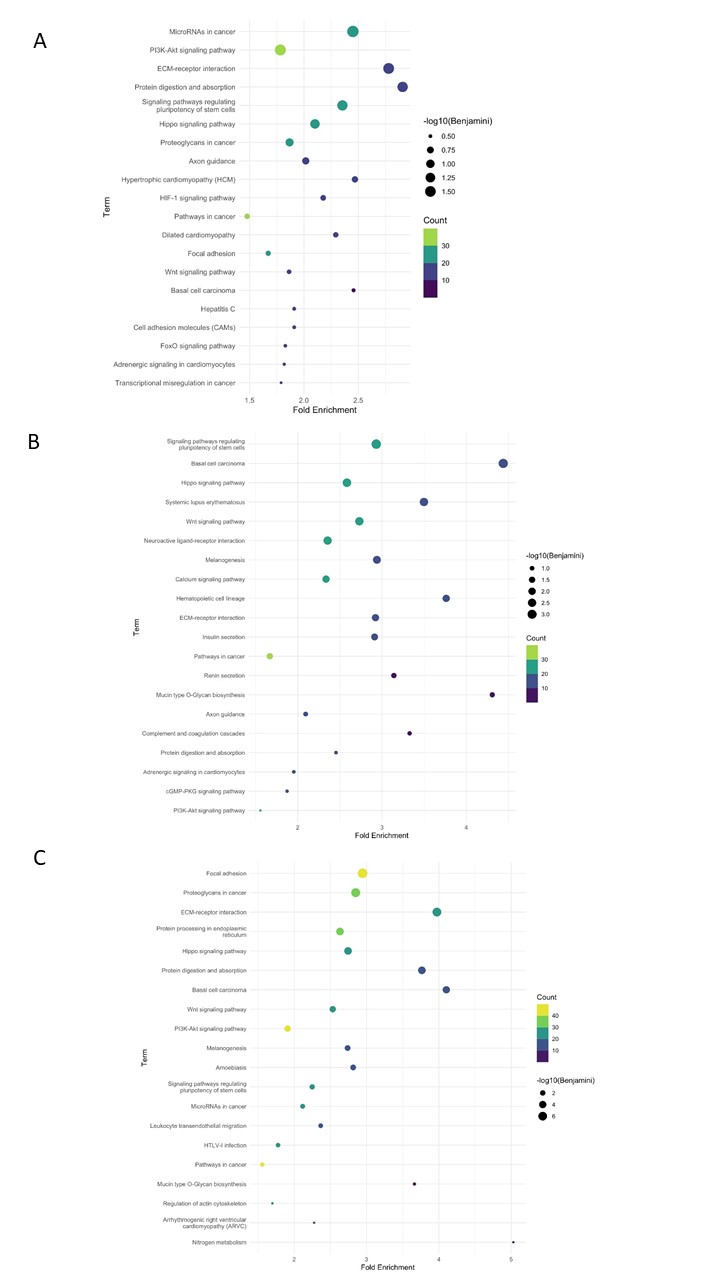

Supplement: Supplementary file 7 — Additional file 7: Fig. S6. Differentially expressed KEGG pathways. A Top 20 enriched KEGG pathways in day 15 dataset. B Top 20 enriched KEGG pathways in day 21 dataset. C Top 20 enriched KEGG pathways in day 34 dataset. Differentially expressed genes used in this analysis were restricted to those with adjusted p value<0.1 and a FC>1.5. Benjamini-Hochberg correction was applied for multiple comparisons. [file 13229_2023_540_MOESM7_ESM.jpg]

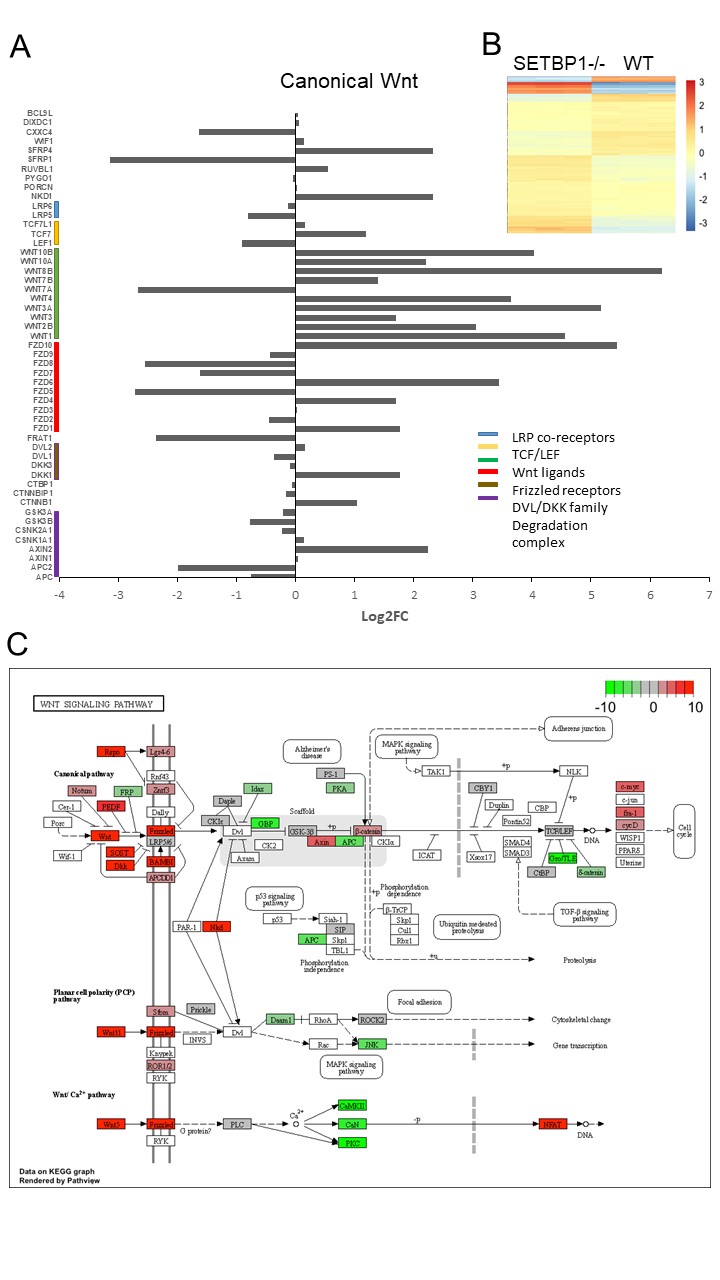

Supplement: Supplementary file 8 — Additional file 8: Fig. S7. Altered expression of genes associated with Wnt pathway at day 34. A Transcriptomic expression of canonical-Wnt related genes represented as Log2FC. B Heatmap depicting differentially expressed transcripts for Wnt signalling (GO:0016055). Differentially regulated transcripts used in this analysis were restricted to those with adjusted p value<0.1, and a FC>1.5. Benjamini-Hochberg correction was applied for multiple comparisons. C WNT signalling pathway graphical representation rendered by Pathview. Image shows up and downregulated pathway components in the SETBP1-/- compared with parental for canonical, planar cell polarity and calcium pathways after intersecting the data with KEGG pathway maps. [file 13229_2023_540_MOESM8_ESM.jpg]

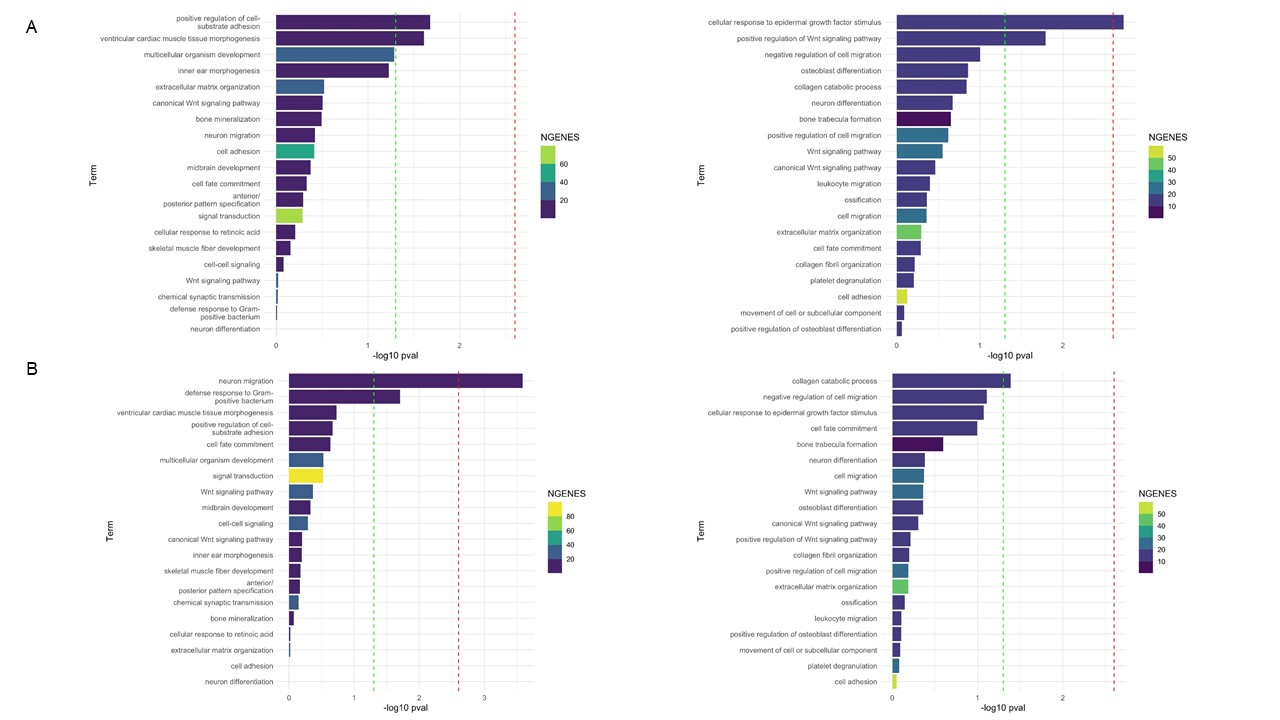

Supplement: Supplementary file 9 — Additional file 9: Fig. S8. MAGMA enrichment results. A Barplots indicating enrichment for common variants associated with ASD at day 21 (left) and day 34 (right). B Barplots indicating enrichment for common variants associated with Intelligence at day 21 (left) and day 34 (right). Green dashed line indicates nominal p value threshold, red dashed line indicates Bonferroni adjusted-p-value threshold for multiple comparisons. [file 13229_2023_540_MOESM9_ESM.jpg]

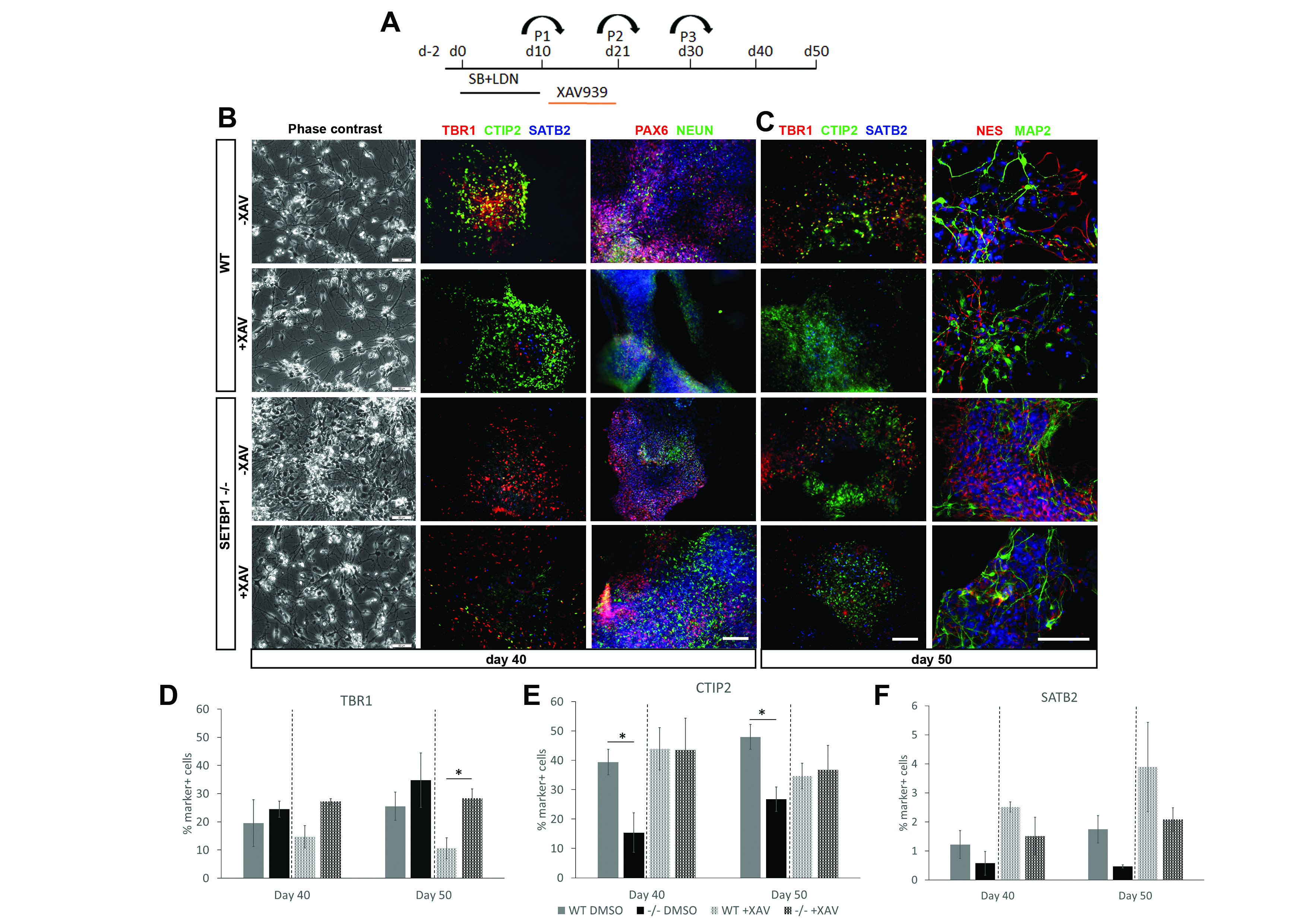

Supplement: Supplementary file 10 — Additional file10: Fig. S9. Phenotype recovery after XAV939 treatment. A Experimental scheme indicating window for XAV939 treatment. B, C Phase contrast and fluorescent images of cultures in basal and XAV condition at day 40 and day 50. The blue staining in the 3rd and 5th column are nuclei stained with DAPI. Scale bar: 100uM. D–F Bar- graphs showing quantification of TBR1, CTIP2 and SATB2 positive neurons from basal and XAV939 treated cultures at day 40 and 50. Data presented as mean ± s.e.m for each genotype with a minimum of three independent experiments carried out per line. Student’s T test was used to compare the expression between the two lines in each condition, Basal cultures: Day 40 TBR1+ cells P=0.714, CTIP2+ cells P=0.03, SATB2+ cells P=0.476; Day 50 TBR1+ cells P=0.390, CTIP2+ cells P=0.035, SATB2+ cells P=0.166. XAV treated cultures: Day 40 TBR1+ cells P=0.177, CTIP2+ cells P=0.979, SATB2+ cells P=0.255; Day 50 TBR1+ cells P=0.016, CTIP2+ cells P=0.830, SATB2+ cells P=0.298. (*p ≤ 0.05). [file 13229_2023_540_MOESM10_ESM.jpg]

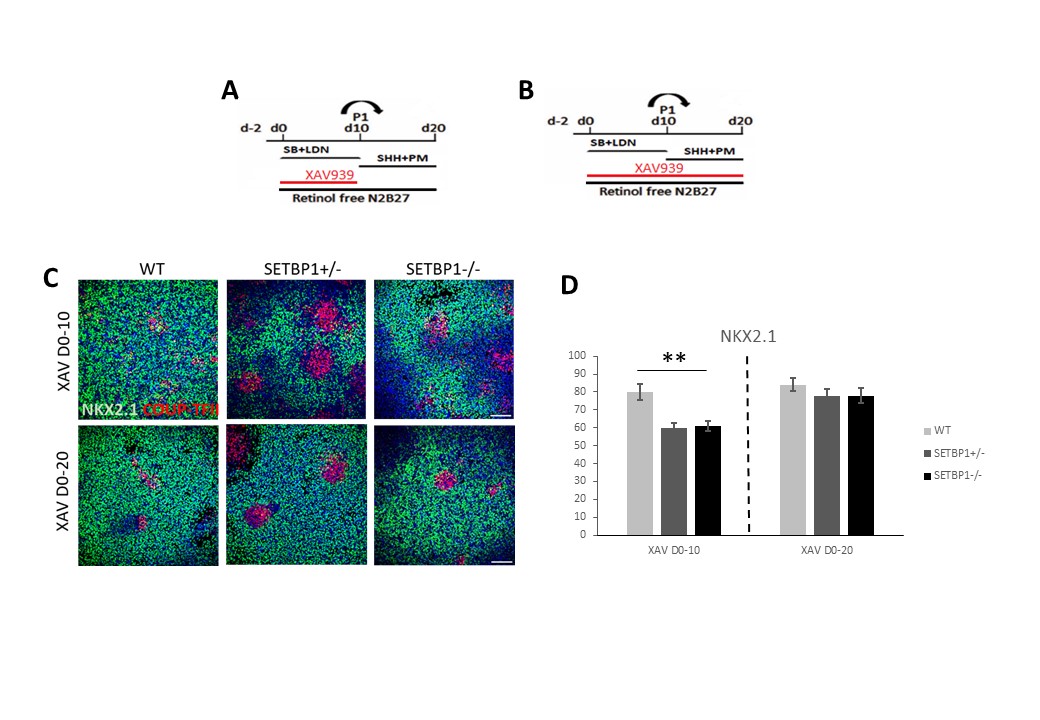

Supplement: Supplementary file 11 — Additional file11: Fig. S10. Effect of extended XAV939 treatment in ventral cultures. A, B Experimental scheme indicating windows for XAV939 treatment in the standard MGE protocol and XAV extended protocol. C Immunostaining of day 20 cultures for NKX2.1 (green) and COUP-TFII (red). D Quantitative data of NKX2.1 marker expression presented as mean ± s.e.m. for each genotype with three biological replicas carried out per line (WT, HET1, HET2, Homo1, and Homo2) E Expression of PAX6 (red). F Expression of GSH2 (red). Dapi was used to label all nuclei. Scale bar: 100uM. [file 13229_2023_540_MOESM11_ESM.jpg]
